# Supplementary figures and images for: Genetic Diversity and Fingerprinting of 231 Mango Germplasm Using Genome SSR Markers (part 2 of 2)
Source: Int J Mol Sci. 2024 Dec 19;25(24):13625. doi: 10.3390/ijms252413625 (PMC11728225; doi:10.3390/ijms252413625)

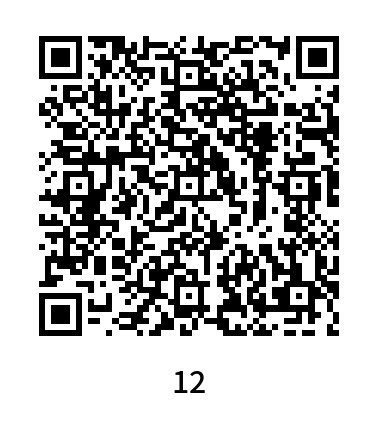

Supplement: Supplementary file 1 [file ijms-25-13625-s001.zip › Figure S1 Fingerprint two-dimensional barcode/two-dimensional code/Name Gundoo Origin or Source Indonesia Fingerprint 33347715111224132444122568226655.png]

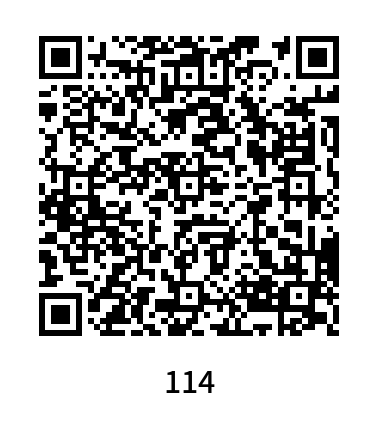

Supplement: Supplementary file 1 [file ijms-25-13625-s001.zip › Figure S1 Fingerprint two-dimensional barcode/two-dimensional code/Name Haden Origin or Source America Fingerprint 36461115132414333424243416264635.png]

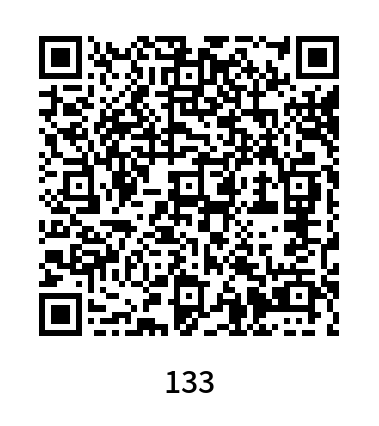

Supplement: Supplementary file 1 [file ijms-25-13625-s001.zip › Figure S1 Fingerprint two-dimensional barcode/two-dimensional code/Name Haibao Origin or Source China Fingerprint 24451525151312121511355658144636.png]

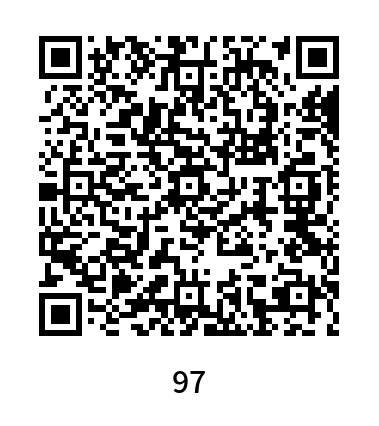

Supplement: Supplementary file 1 [file ijms-25-13625-s001.zip › Figure S1 Fingerprint two-dimensional barcode/two-dimensional code/Name Herman Origin or Source America Fingerprint 26457755563444342344231468566656.png]

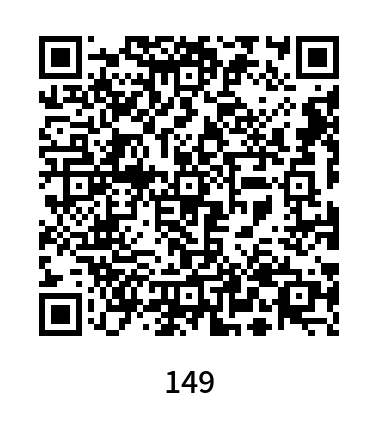

Supplement: Supplementary file 1 [file ijms-25-13625-s001.zip › Figure S1 Fingerprint two-dimensional barcode/two-dimensional code/Name Hongjingfeng Origin or Source ChinaTaiwan Fingerprint 25461558562312143314233516144635.png]

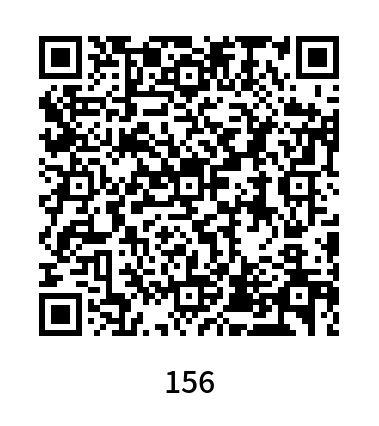

Supplement: Supplementary file 1 [file ijms-25-13625-s001.zip › Figure S1 Fingerprint two-dimensional barcode/two-dimensional code/Name Hongjinlong Origin or Source ChinaTaiwan Fingerprint 45561558362315132314343516244535.png]

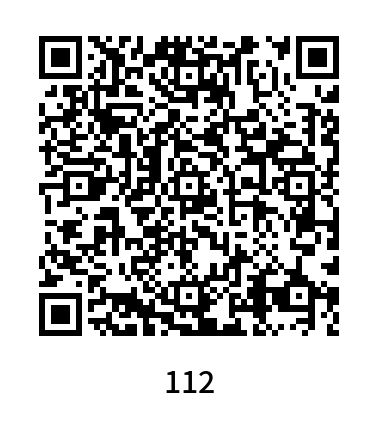

Supplement: Supplementary file 1 [file ijms-25-13625-s001.zip › Figure S1 Fingerprint two-dimensional barcode/two-dimensional code/Name Hongmang No.10 Origin or Source America Fingerprint 24464555131225231315342615163445.png]

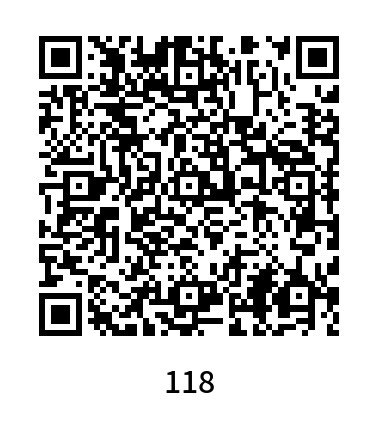

Supplement: Supplementary file 1 [file ijms-25-13625-s001.zip › Figure S1 Fingerprint two-dimensional barcode/two-dimensional code/Name Hongmang No.11 Origin or Source America Fingerprint 45461655132311133415342368265634.png]

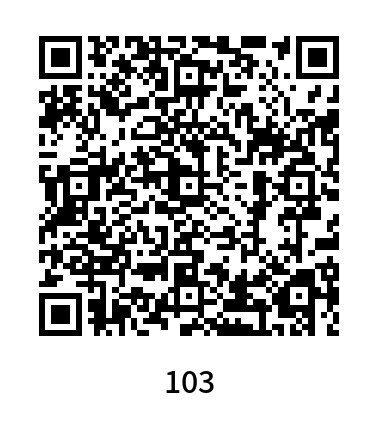

Supplement: Supplementary file 1 [file ijms-25-13625-s001.zip › Figure S1 Fingerprint two-dimensional barcode/two-dimensional code/Name Hongmang No.8 Origin or Source America Fingerprint 26461518361215342314242315664435.png]

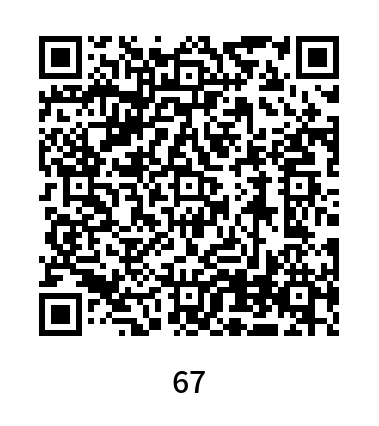

Supplement: Supplementary file 1 [file ijms-25-13625-s001.zip › Figure S1 Fingerprint two-dimensional barcode/two-dimensional code/Name Hongmangguo Origin or Source America Fingerprint 35361115562424133444242316224635.png]

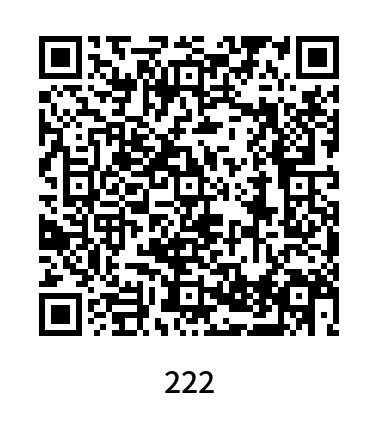

Supplement: Supplementary file 1 [file ijms-25-13625-s001.zip › Figure S1 Fingerprint two-dimensional barcode/two-dimensional code/Name Hongwacheng Origin or Source China Fingerprint 25361428562412141355235666144656.png]

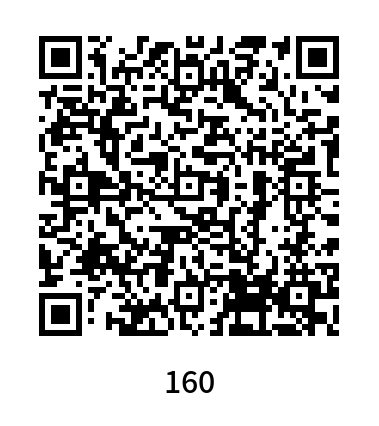

Supplement: Supplementary file 1 [file ijms-25-13625-s001.zip › Figure S1 Fingerprint two-dimensional barcode/two-dimensional code/Name Hongxiangya R Origin or Source China Fingerprint 22486618152345343344242611565645.png]

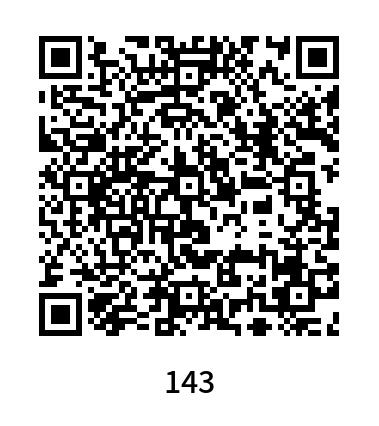

Supplement: Supplementary file 1 [file ijms-25-13625-s001.zip › Figure S1 Fingerprint two-dimensional barcode/two-dimensional code/Name Huangxiangya Origin or Source China Fingerprint 25561118333312143355235666144656.png]

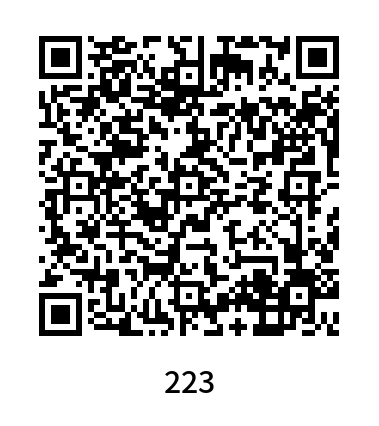

Supplement: Supplementary file 1 [file ijms-25-13625-s001.zip › Figure S1 Fingerprint two-dimensional barcode/two-dimensional code/Name Huangxing Origin or Source China Fingerprint 22455655151345333515234656564645.png]

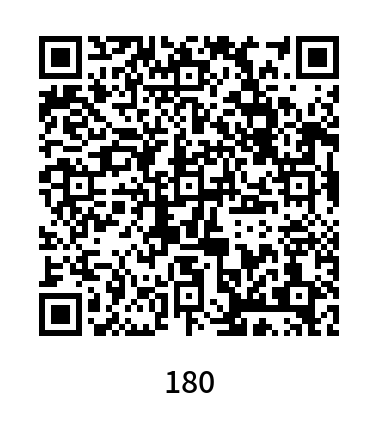

Supplement: Supplementary file 1 [file ijms-25-13625-s001.zip › Figure S1 Fingerprint two-dimensional barcode/two-dimensional code/Name Huangyu Origin or Source Thailand Fingerprint 25451628153412142315235635144636.png]

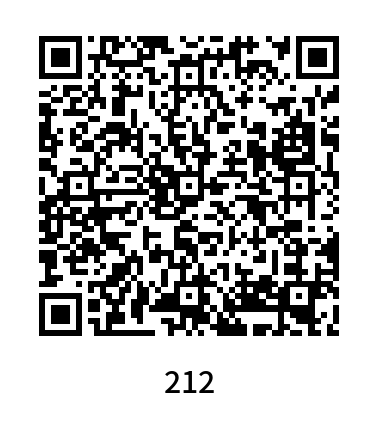

Supplement: Supplementary file 1 [file ijms-25-13625-s001.zip › Figure S1 Fingerprint two-dimensional barcode/two-dimensional code/Name Hubaoya Origin or Source China Fingerprint 22465558553415243515135656233433.png]

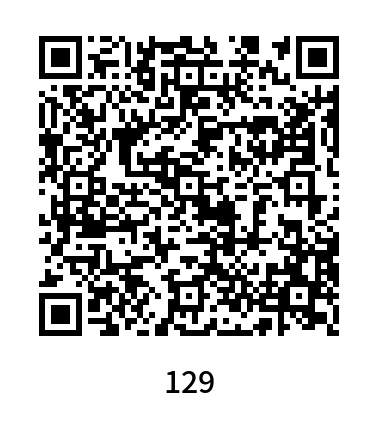

Supplement: Supplementary file 1 [file ijms-25-13625-s001.zip › Figure S1 Fingerprint two-dimensional barcode/two-dimensional code/Name Hutou Origin or Source China Fingerprint 22464468152214443355342318253636.png]

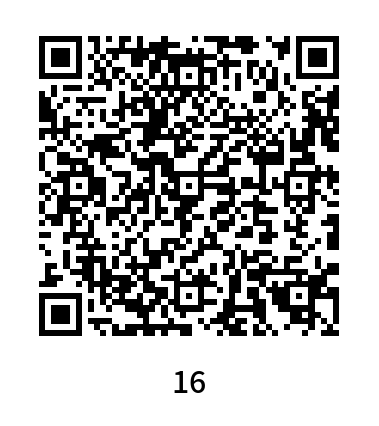

Supplement: Supplementary file 1 [file ijms-25-13625-s001.zip › Figure S1 Fingerprint two-dimensional barcode/two-dimensional code/Name Indonesia No.1 Origin or Source Indonesia Fingerprint 25681617162415133444120016455645.png]

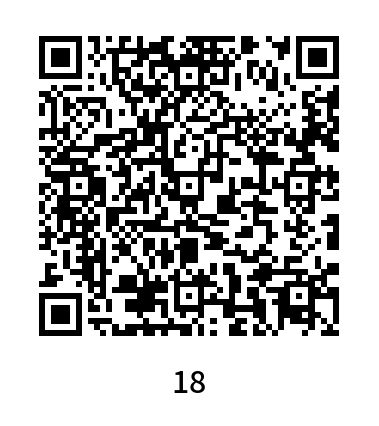

Supplement: Supplementary file 1 [file ijms-25-13625-s001.zip › Figure S1 Fingerprint two-dimensional barcode/two-dimensional code/Name Indonesia No.2 Origin or Source Indonesia Fingerprint 36467715132414333414243416264635.png]

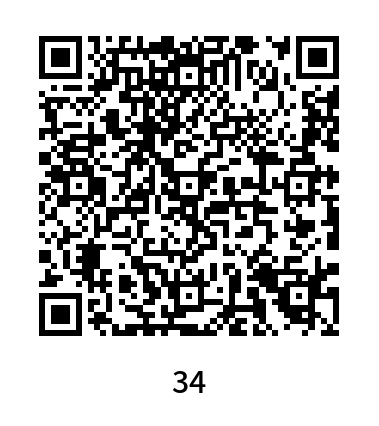

Supplement: Supplementary file 1 [file ijms-25-13625-s001.zip › Figure S1 Fingerprint two-dimensional barcode/two-dimensional code/Name Indonesia No.3 Origin or Source Indonesia Fingerprint 22455658152444141344344468164656.png]

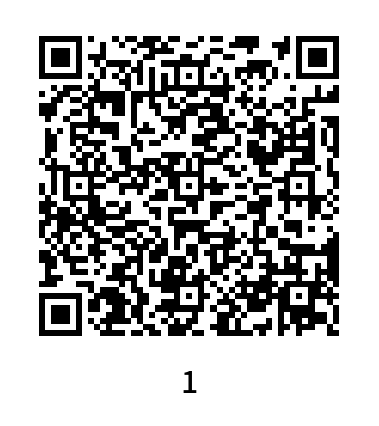

Supplement: Supplementary file 1 [file ijms-25-13625-s001.zip › Figure S1 Fingerprint two-dimensional barcode/two-dimensional code/Name Irwin Origin or Source America Fingerprint 35667755562411133444242411264555.png]

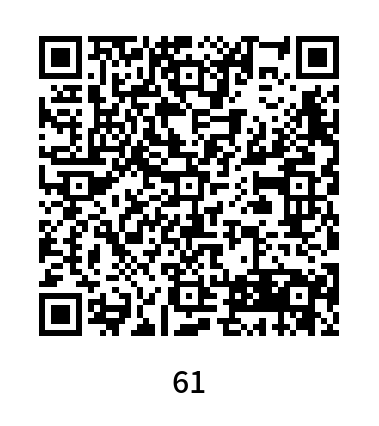

Supplement: Supplementary file 1 [file ijms-25-13625-s001.zip › Figure S1 Fingerprint two-dimensional barcode/two-dimensional code/Name Jianmang Origin or Source Cambodia Fingerprint 24455558131215141315335516163445.png]

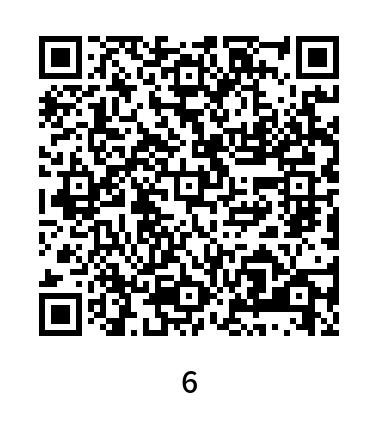

Supplement: Supplementary file 1 [file ijms-25-13625-s001.zip › Figure S1 Fingerprint two-dimensional barcode/two-dimensional code/Name JinHuang Origin or Source ChinaTaiwan Fingerprint 45341555132445232355334615264635.png]

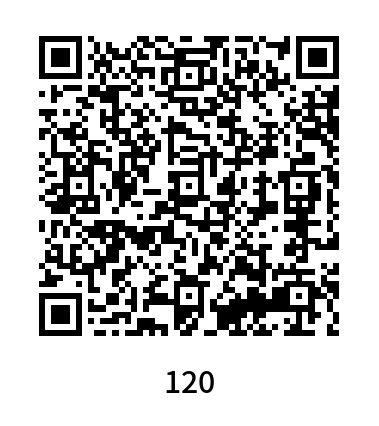

Supplement: Supplementary file 1 [file ijms-25-13625-s001.zip › Figure S1 Fingerprint two-dimensional barcode/two-dimensional code/Name Jinsui Origin or Source China Fingerprint 25464557132315341335245618244646.png]

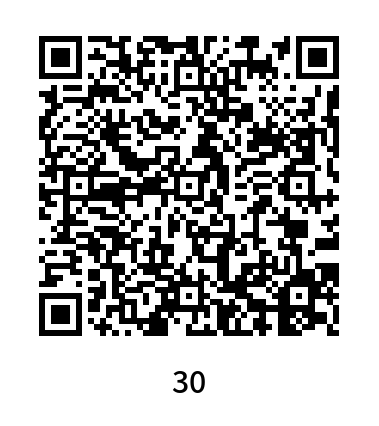

Supplement: Supplementary file 1 [file ijms-25-13625-s001.zip › Figure S1 Fingerprint two-dimensional barcode/two-dimensional code/Name Julie Origin or Source the West Indies Fingerprint 57361455362315231414122213443635.png]

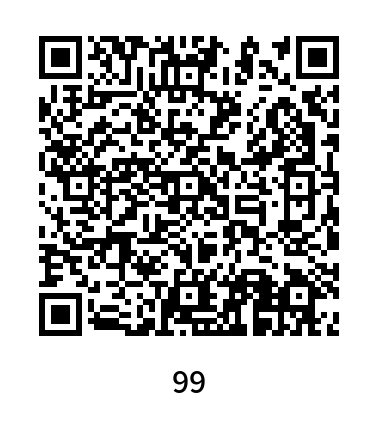

Supplement: Supplementary file 1 [file ijms-25-13625-s001.zip › Figure S1 Fingerprint two-dimensional barcode/two-dimensional code/Name Kasturi Origin or Source Indonesia Fingerprint 22256638232213231345133325441613.png]

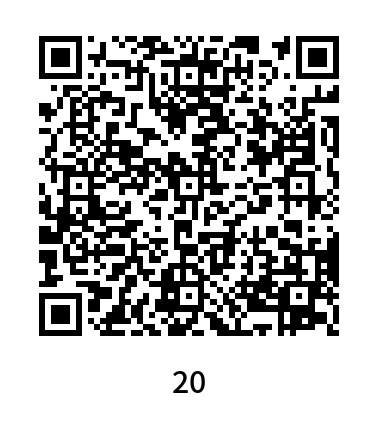

Supplement: Supplementary file 1 [file ijms-25-13625-s001.zip › Figure S1 Fingerprint two-dimensional barcode/two-dimensional code/Name Keitt Origin or Source America Fingerprint 35461155262324332444244511254615.png]

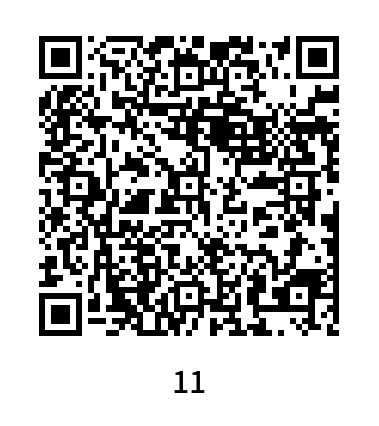

Supplement: Supplementary file 1 [file ijms-25-13625-s001.zip › Figure S1 Fingerprint two-dimensional barcode/two-dimensional code/Name KenSington Origin or Source Australia Fingerprint 46366635152614133415232515256656.png]

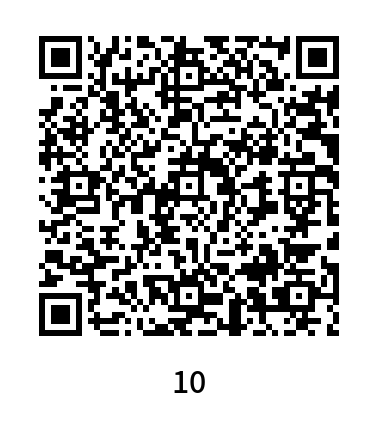

Supplement: Supplementary file 1 [file ijms-25-13625-s001.zip › Figure S1 Fingerprint two-dimensional barcode/two-dimensional code/Name Kent Origin or Source America Fingerprint 35347715152424332315344518226635.png]

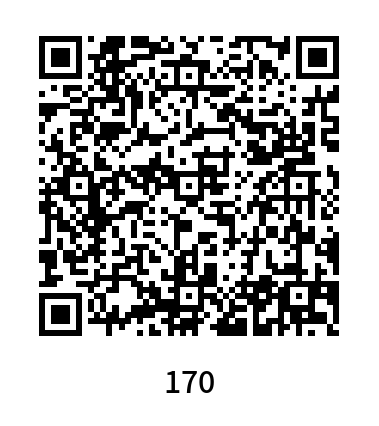

Supplement: Supplementary file 1 [file ijms-25-13625-s001.zip › Figure S1 Fingerprint two-dimensional barcode/two-dimensional code/Name KRS Origin or Source Australia Fingerprint 46366635562614133415232515254656.png]

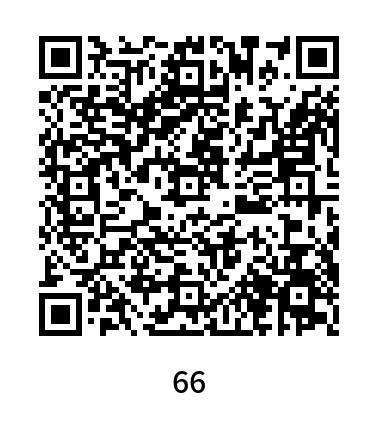

Supplement: Supplementary file 1 [file ijms-25-13625-s001.zip › Figure S1 Fingerprint two-dimensional barcode/two-dimensional code/Name kwini Origin or Source Indonesia Fingerprint 33361115354414133444243411224555.png]

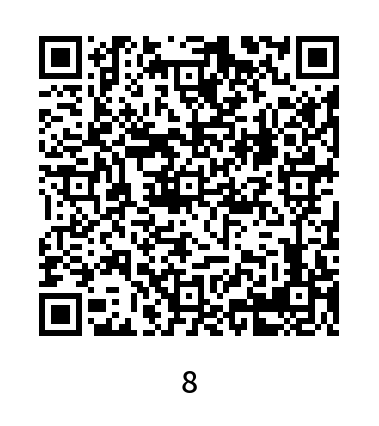

Supplement: Supplementary file 1 [file ijms-25-13625-s001.zip › Figure S1 Fingerprint two-dimensional barcode/two-dimensional code/Name kyo savoy Origin or Source Thailand Fingerprint 25681118113355333434222518465646.png]

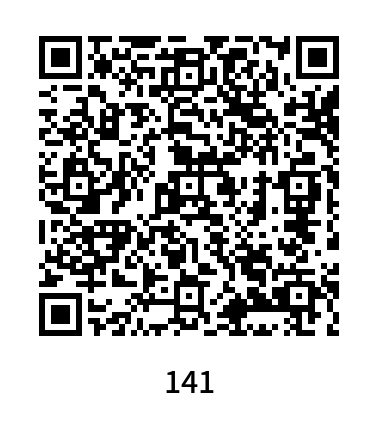

Supplement: Supplementary file 1 [file ijms-25-13625-s001.zip › Figure S1 Fingerprint two-dimensional barcode/two-dimensional code/Name Langra Origin or Source India Fingerprint 24485655373414141411234568156656.png]

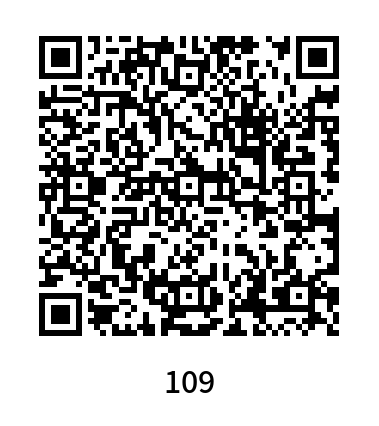

Supplement: Supplementary file 1 [file ijms-25-13625-s001.zip › Figure S1 Fingerprint two-dimensional barcode/two-dimensional code/Name Lengjingdamang Origin or Source China Fingerprint 25685615353444113414232213225635.png]

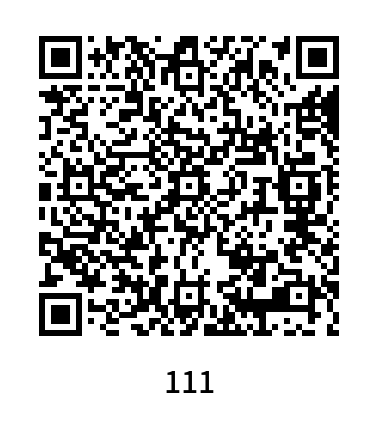

Supplement: Supplementary file 1 [file ijms-25-13625-s001.zip › Figure S1 Fingerprint two-dimensional barcode/two-dimensional code/Name Lilley Origin or Source America Fingerprint 25561111132312141344343544244656.png]

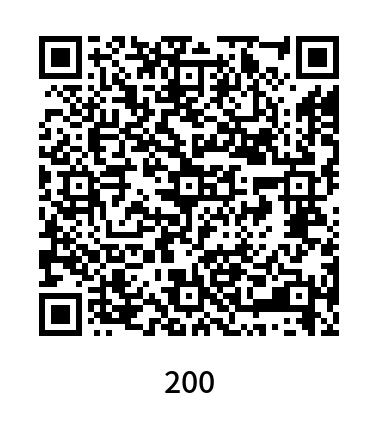

Supplement: Supplementary file 1 [file ijms-25-13625-s001.zip › Figure S1 Fingerprint two-dimensional barcode/two-dimensional code/Name Linsheng Origin or Source China Fingerprint 22475558353414123413242437465535.png]

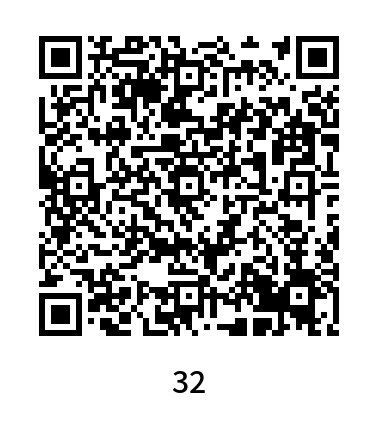

Supplement: Supplementary file 1 [file ijms-25-13625-s001.zip › Figure S1 Fingerprint two-dimensional barcode/two-dimensional code/Name Lippens Origin or Source America Fingerprint 56461155152414133344242318254556.png]

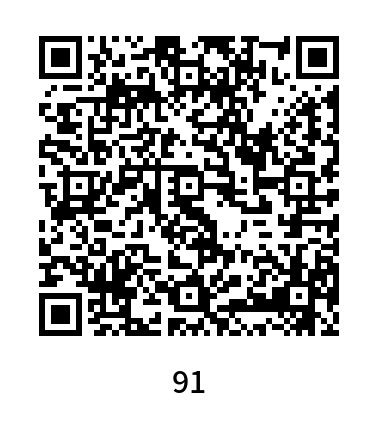

Supplement: Supplementary file 1 [file ijms-25-13625-s001.zip › Figure S1 Fingerprint two-dimensional barcode/two-dimensional code/Name Liuxiang Origin or Source Singapore Fingerprint 24445577361225142315453656153336.png]

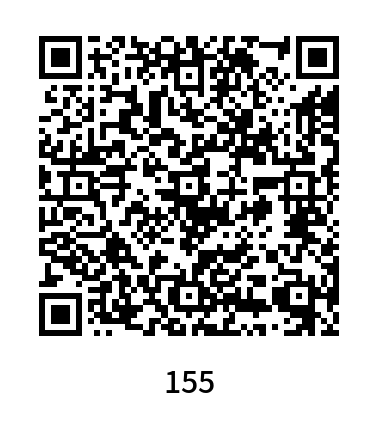

Supplement: Supplementary file 1 [file ijms-25-13625-s001.zip › Figure S1 Fingerprint two-dimensional barcode/two-dimensional code/Name Longjing Origin or Source China Fingerprint 25561117353312142344235616144656.png]

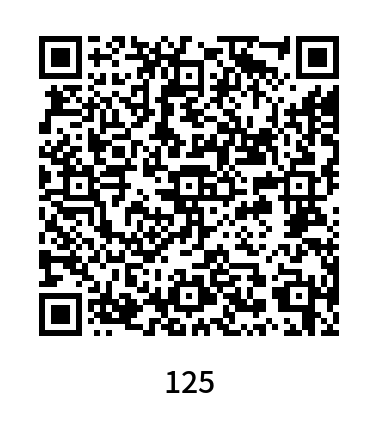

Supplement: Supplementary file 1 [file ijms-25-13625-s001.zip › Figure S1 Fingerprint two-dimensional barcode/two-dimensional code/Name Lvpimang Origin or Source China Fingerprint 25664435352314343415243568263635.png]

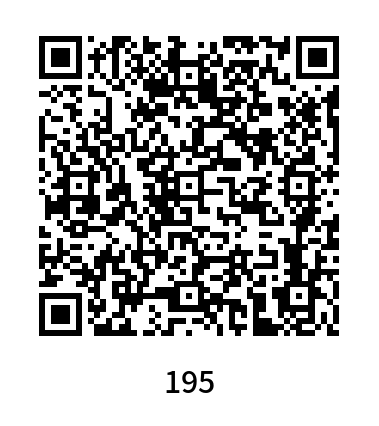

Supplement: Supplementary file 1 [file ijms-25-13625-s001.zip › Figure S1 Fingerprint two-dimensional barcode/two-dimensional code/Name Lvsong 20 Origin or Source Thailand Fingerprint 24341868352325112355455656113436.png]

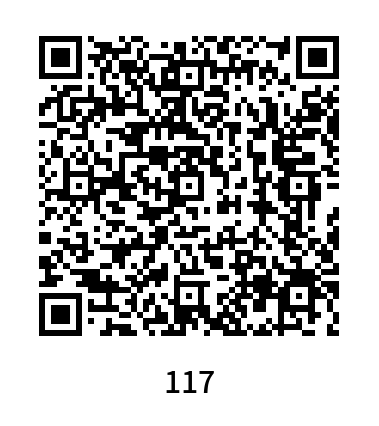

Supplement: Supplementary file 1 [file ijms-25-13625-s001.zip › Figure S1 Fingerprint two-dimensional barcode/two-dimensional code/Name Lvsong Origin or Source Thailand Fingerprint 24451458561325132345455656113436.png]

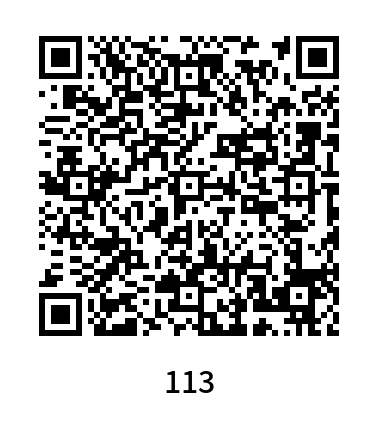

Supplement: Supplementary file 1 [file ijms-25-13625-s001.zip › Figure S1 Fingerprint two-dimensional barcode/two-dimensional code/Name Macheso Origin or Source Myanmar Fingerprint 276A5555112344131314345513344636.png]

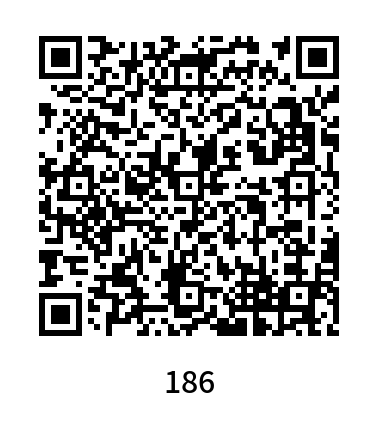

Supplement: Supplementary file 1 [file ijms-25-13625-s001.zip › Figure S1 Fingerprint two-dimensional barcode/two-dimensional code/Name magovar Origin or Source India Fingerprint 25481515332311111311255558263656.png]

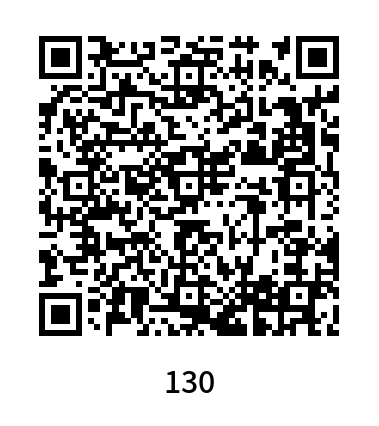

Supplement: Supplementary file 1 [file ijms-25-13625-s001.zip › Figure S1 Fingerprint two-dimensional barcode/two-dimensional code/Name Mallika Origin or Source India Fingerprint 25664415333314343315233516463635.png]

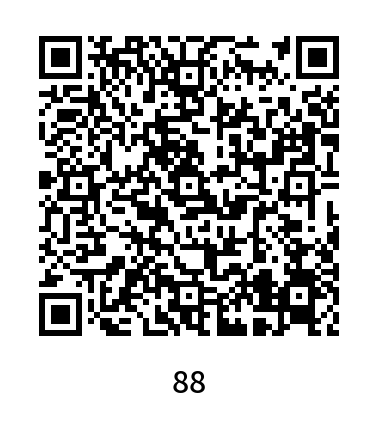

Supplement: Supplementary file 1 [file ijms-25-13625-s001.zip › Figure S1 Fingerprint two-dimensional barcode/two-dimensional code/Name Manzano Origin or Source America Fingerprint 35667755133311343444244568243456.png]

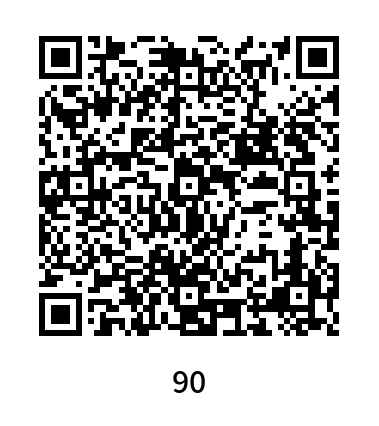

Supplement: Supplementary file 1 [file ijms-25-13625-s001.zip › Figure S1 Fingerprint two-dimensional barcode/two-dimensional code/Name Miami late Origin or Source America Fingerprint 56661115132314133414342411224635.png]

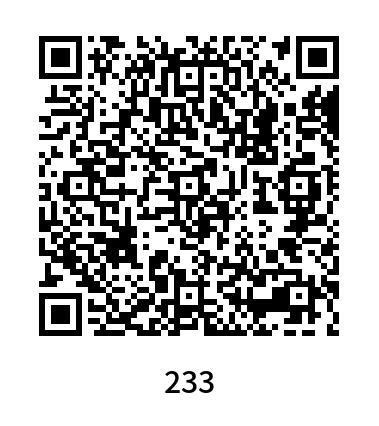

Supplement: Supplementary file 1 [file ijms-25-13625-s001.zip › Figure S1 Fingerprint two-dimensional barcode/two-dimensional code/Name Mian 8 Origin or Source Myanmar Fingerprint 25465515112244132335233578673656.png]

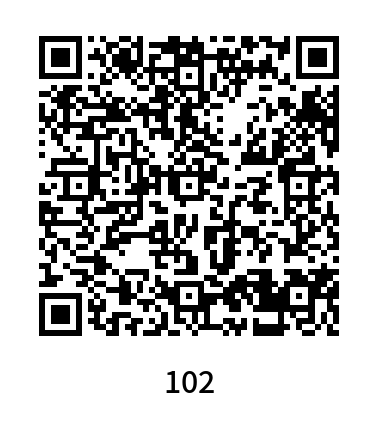

Supplement: Supplementary file 1 [file ijms-25-13625-s001.zip › Figure S1 Fingerprint two-dimensional barcode/two-dimensional code/Name Myahintha Origin or Source Myanmar Fingerprint 23351655133414231314231518456656.png]

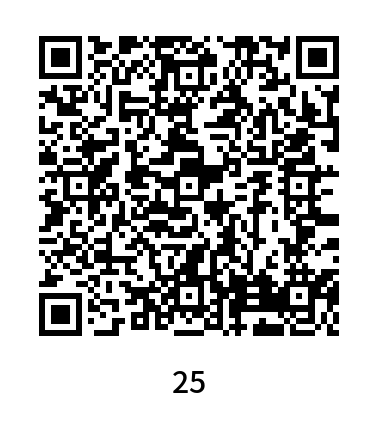

Supplement: Supplementary file 1 [file ijms-25-13625-s001.zip › Figure S1 Fingerprint two-dimensional barcode/two-dimensional code/Name Mylepania Origin or Source Australia Fingerprint 46364635352314131415234515456656.png]

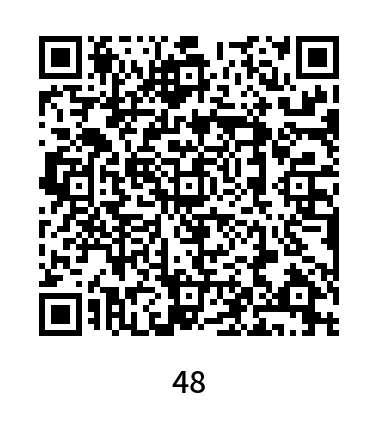

Supplement: Supplementary file 1 [file ijms-25-13625-s001.zip › Figure S1 Fingerprint two-dimensional barcode/two-dimensional code/Name Nam Dok Mai Sitong Origin or Source Thailand Fingerprint 25461168152315333511245618266656.png]

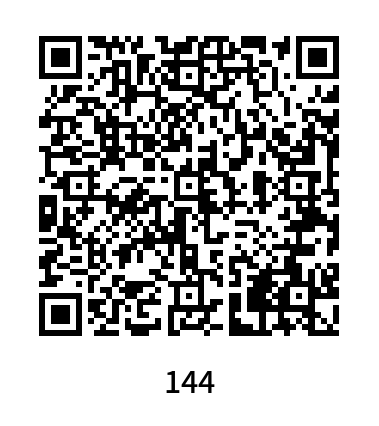

Supplement: Supplementary file 1 [file ijms-25-13625-s001.zip › Figure S1 Fingerprint two-dimensional barcode/two-dimensional code/Name Nam Klang Wan Origin or Source Thailand Fingerprint 24445558112255232315343658662434.png]

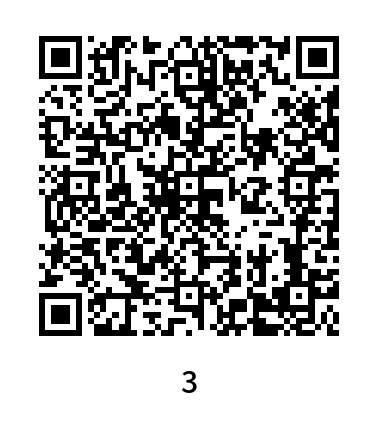

Supplement: Supplementary file 1 [file ijms-25-13625-s001.zip › Figure S1 Fingerprint two-dimensional barcode/two-dimensional code/Name NamDocMai Origin or Source Thailand Fingerprint 24444555161225231315345615164445.png]

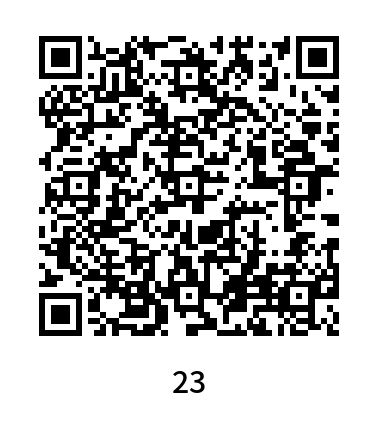

Supplement: Supplementary file 1 [file ijms-25-13625-s001.zip › Figure S1 Fingerprint two-dimensional barcode/two-dimensional code/Name NamDocMai4 Origin or Source Thailand Fingerprint 25461168552315331315245618266656.png]

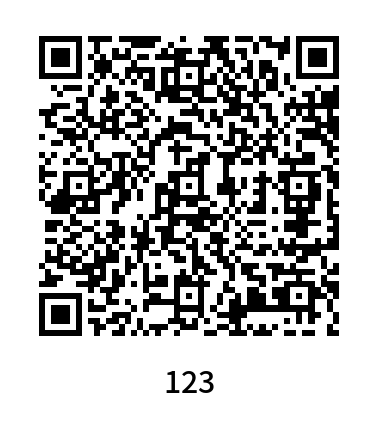

Supplement: Supplementary file 1 [file ijms-25-13625-s001.zip › Figure S1 Fingerprint two-dimensional barcode/two-dimensional code/Name Neelum Origin or Source India Fingerprint 55664415152311333423255518466656.png]

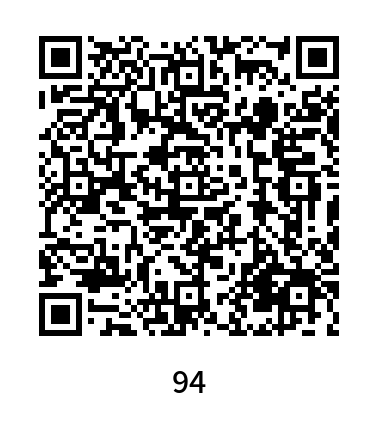

Supplement: Supplementary file 1 [file ijms-25-13625-s001.zip › Figure S1 Fingerprint two-dimensional barcode/two-dimensional code/Name Okrang Origin or Source Thailand Fingerprint 22464458562214441355342318253636.png]

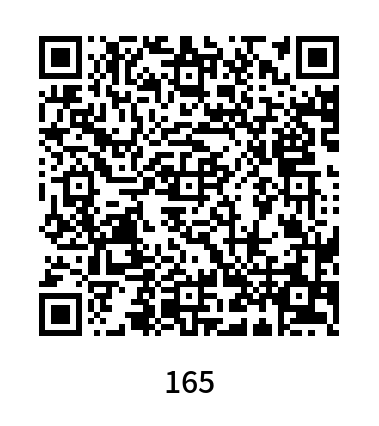

Supplement: Supplementary file 1 [file ijms-25-13625-s001.zip › Figure S1 Fingerprint two-dimensional barcode/two-dimensional code/Name Ono Origin or Source America Fingerprint 35561128133414133444232516263656.png]

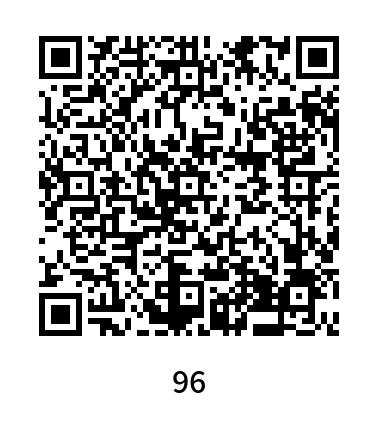

Supplement: Supplementary file 1 [file ijms-25-13625-s001.zip › Figure S1 Fingerprint two-dimensional barcode/two-dimensional code/Name Pairi 905 Origin or Source India Fingerprint 22345625132414112311232467563656.png]

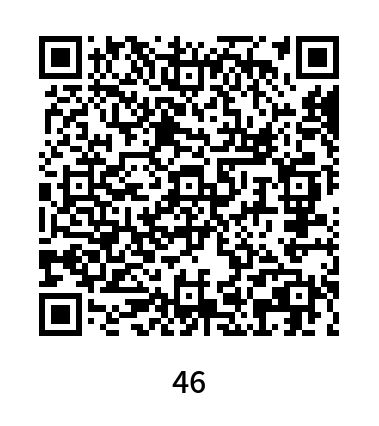

Supplement: Supplementary file 1 [file ijms-25-13625-s001.zip › Figure S1 Fingerprint two-dimensional barcode/two-dimensional code/Name Palmer Origin or Source America Fingerprint 35347715562324132414443516226635.png]

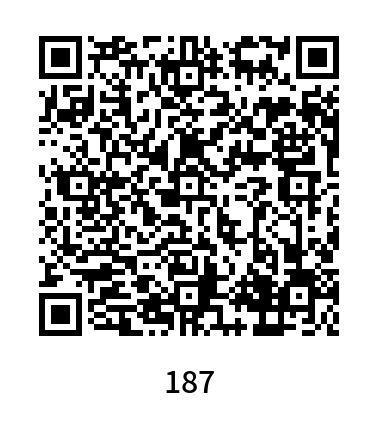

Supplement: Supplementary file 1 [file ijms-25-13625-s001.zip › Figure S1 Fingerprint two-dimensional barcode/two-dimensional code/Name Panxihong Origin or Source China Fingerprint 22445658562214142355343518163656.png]

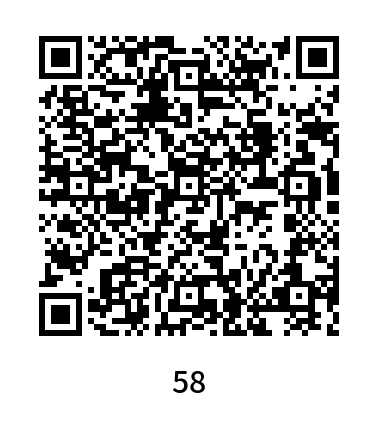

Supplement: Supplementary file 1 [file ijms-25-13625-s001.zip › Figure S1 Fingerprint two-dimensional barcode/two-dimensional code/Name Panyu No.2 Origin or Source China Fingerprint 22465555152445332311235656664645.png]

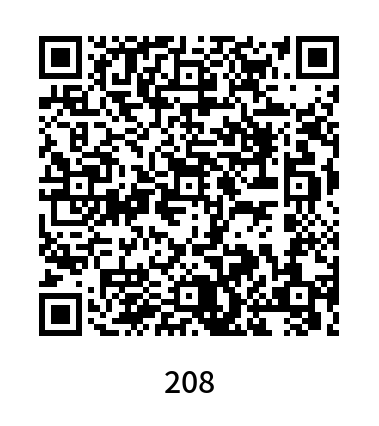

Supplement: Supplementary file 1 [file ijms-25-13625-s001.zip › Figure S1 Fingerprint two-dimensional barcode/two-dimensional code/Name Panyu No.3 Origin or Source China Fingerprint 23455515111425333415342611255645.png]

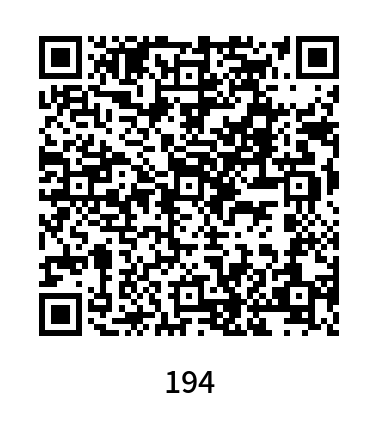

Supplement: Supplementary file 1 [file ijms-25-13625-s001.zip › Figure S1 Fingerprint two-dimensional barcode/two-dimensional code/Name Panyu No.4 Origin or Source China Fingerprint 23341628351344143444342311256645.png]

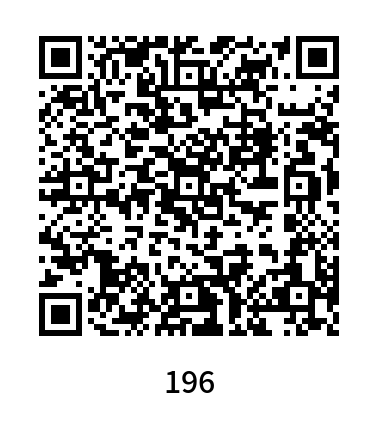

Supplement: Supplementary file 1 [file ijms-25-13625-s001.zip › Figure S1 Fingerprint two-dimensional barcode/two-dimensional code/Name Panyu No.5 Origin or Source China Fingerprint 35581155153444332444342516254635.png]

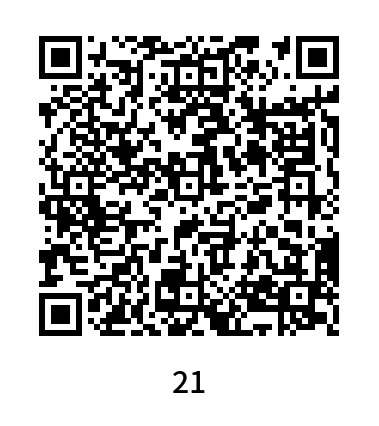

Supplement: Supplementary file 1 [file ijms-25-13625-s001.zip › Figure S1 Fingerprint two-dimensional barcode/two-dimensional code/Name Peach Origin or Source America Fingerprint 26457715112414133414341417254656.png]

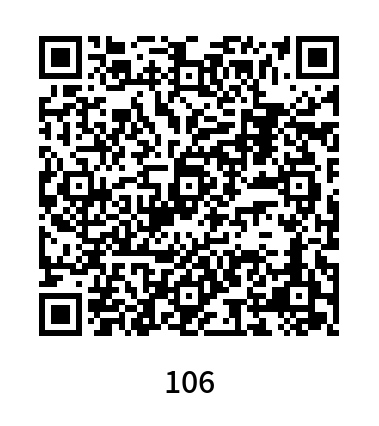

Supplement: Supplementary file 1 [file ijms-25-13625-s001.zip › Figure S1 Fingerprint two-dimensional barcode/two-dimensional code/Name Pills bury Origin or Source America Fingerprint 26561645242544131311344515245656.png]

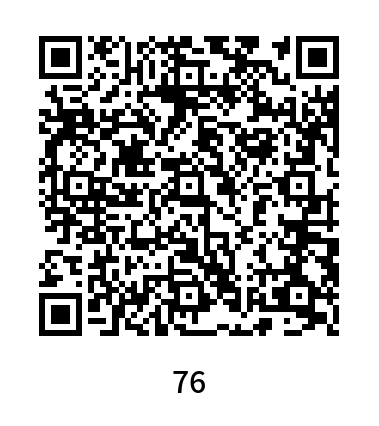

Supplement: Supplementary file 1 [file ijms-25-13625-s001.zip › Figure S1 Fingerprint two-dimensional barcode/two-dimensional code/Name Putao Origin or Source China Fingerprint 234A6639151213241215331616224433.png]

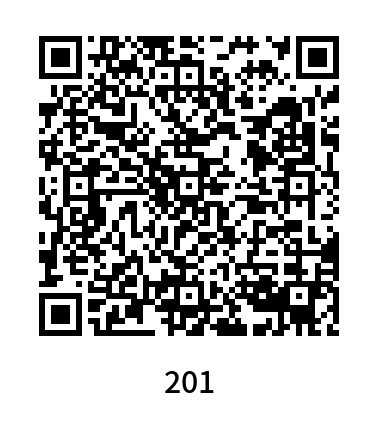

Supplement: Supplementary file 1 [file ijms-25-13625-s001.zip › Figure S1 Fingerprint two-dimensional barcode/two-dimensional code/Name Qiuhong Origin or Source China Fingerprint 22455515562414123423242268465535.png]

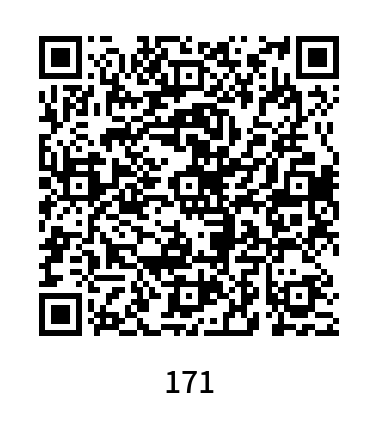

Supplement: Supplementary file 1 [file ijms-25-13625-s001.zip › Figure S1 Fingerprint two-dimensional barcode/two-dimensional code/Name R2E2 Origin or Source Australia Fingerprint 56341615362424132315234511255656.png]

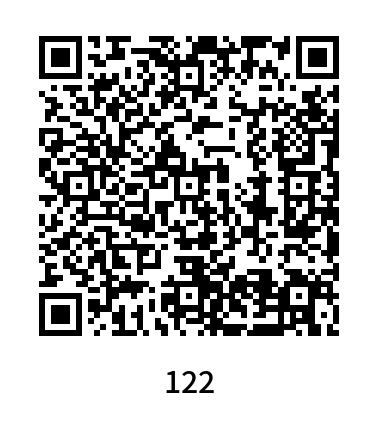

Supplement: Supplementary file 1 [file ijms-25-13625-s001.zip › Figure S1 Fingerprint two-dimensional barcode/two-dimensional code/Name Renong No.2 Origin or Source China Fingerprint 25661155132311331314124511265655.png]

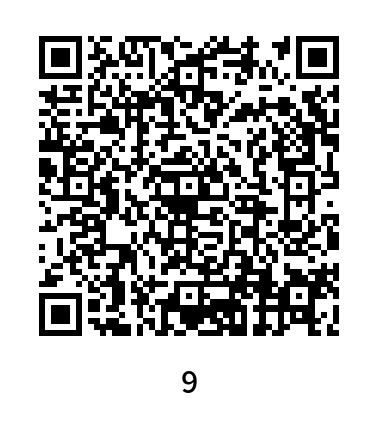

Supplement: Supplementary file 1 [file ijms-25-13625-s001.zip › Figure S1 Fingerprint two-dimensional barcode/two-dimensional code/Name Renong1 Origin or Source Australia Fingerprint 36661613132212113444232215256656.png]

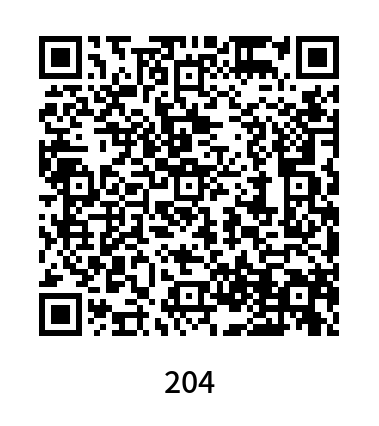

Supplement: Supplementary file 1 [file ijms-25-13625-s001.zip › Figure S1 Fingerprint two-dimensional barcode/two-dimensional code/Name Repin No.16 Origin or Source China Fingerprint 23561617133314133411344516264656.png]

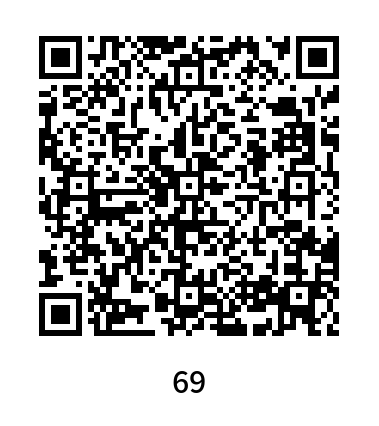

Supplement: Supplementary file 1 [file ijms-25-13625-s001.zip › Figure S1 Fingerprint two-dimensional barcode/two-dimensional code/Name Rjs000l Origin or Source China Fingerprint 22456628571255243415454516253334.png]

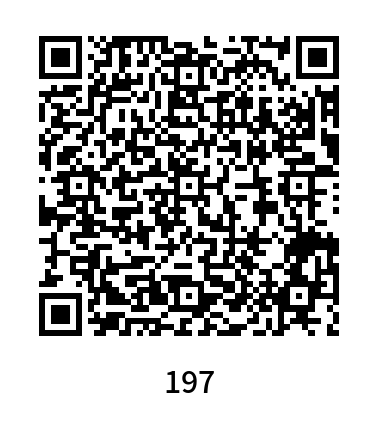

Supplement: Supplementary file 1 [file ijms-25-13625-s001.zip › Figure S1 Fingerprint two-dimensional barcode/two-dimensional code/Name Rosa Origin or Source Brazil Fingerprint 25361115161414141311344416223635.png]

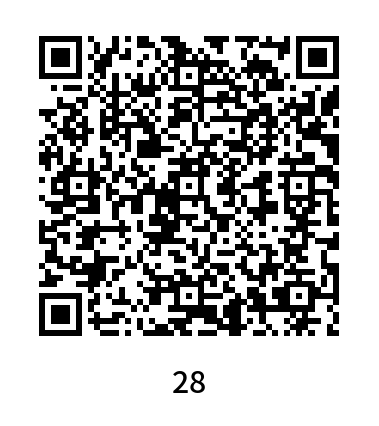

Supplement: Supplementary file 1 [file ijms-25-13625-s001.zip › Figure S1 Fingerprint two-dimensional barcode/two-dimensional code/Name Ruby Origin or Source America Fingerprint 35681111354414333444122411263655.png]

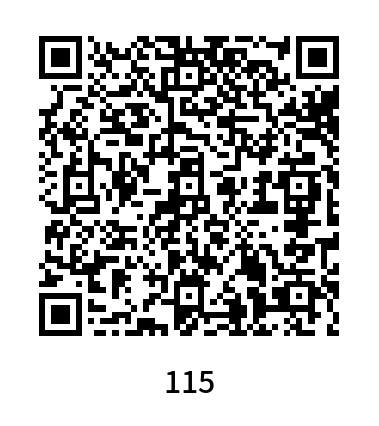

Supplement: Supplementary file 1 [file ijms-25-13625-s001.zip › Figure S1 Fingerprint two-dimensional barcode/two-dimensional code/Name Rumang Origin or Source China Fingerprint 36461115112414133414243416264635.png]

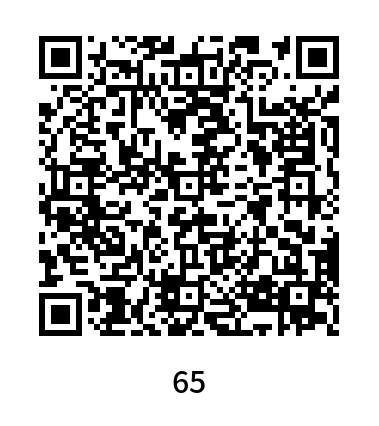

Supplement: Supplementary file 1 [file ijms-25-13625-s001.zip › Figure S1 Fingerprint two-dimensional barcode/two-dimensional code/Name Saber Origin or Source America Fingerprint 25463438562414132314233519251335.png]

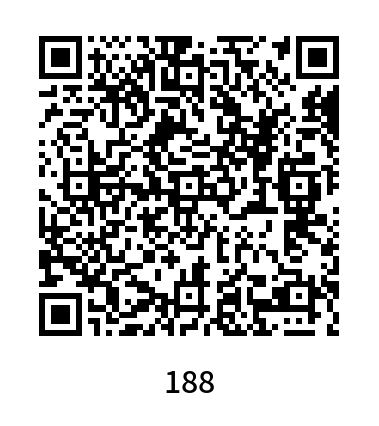

Supplement: Supplementary file 1 [file ijms-25-13625-s001.zip › Figure S1 Fingerprint two-dimensional barcode/two-dimensional code/Name Saigon Origin or Source Vietnam Fingerprint 22685658132415242311342556123435.png]

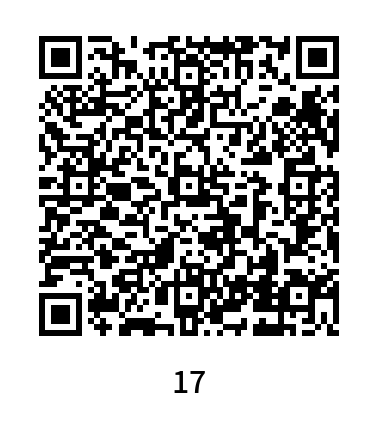

Supplement: Supplementary file 1 [file ijms-25-13625-s001.zip › Figure S1 Fingerprint two-dimensional barcode/two-dimensional code/Name Sandersha Origin or Source America Fingerprint 56681615364414332314242411563455.png]

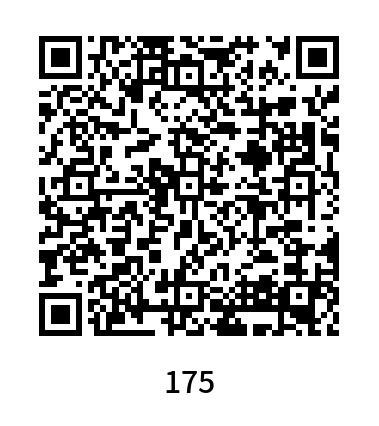

Supplement: Supplementary file 1 [file ijms-25-13625-s001.zip › Figure S1 Fingerprint two-dimensional barcode/two-dimensional code/Name Sannian Origin or Source China Fingerprint 24464555151225132515122336664634.png]

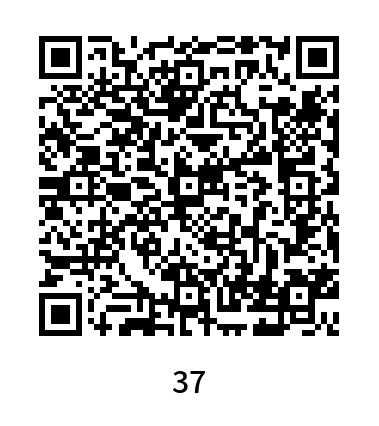

Supplement: Supplementary file 1 [file ijms-25-13625-s001.zip › Figure S1 Fingerprint two-dimensional barcode/two-dimensional code/Name Sensation Origin or Source America Fingerprint 35361115562424133444242316224655.png]

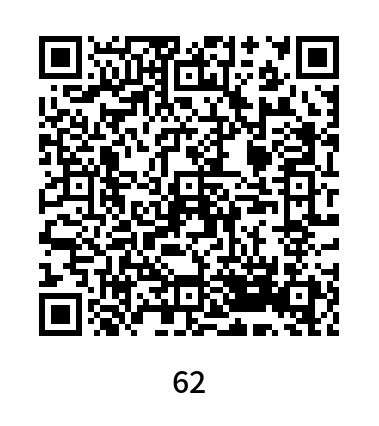

Supplement: Supplementary file 1 [file ijms-25-13625-s001.zip › Figure S1 Fingerprint two-dimensional barcode/two-dimensional code/Name Shanlin Origin or Source ChinaTaiwan Fingerprint 35661611564414131314244411254555.png]

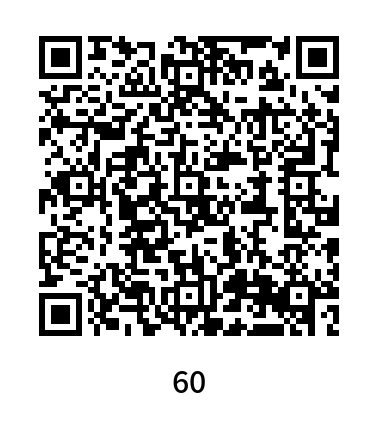

Supplement: Supplementary file 1 [file ijms-25-13625-s001.zip › Figure S1 Fingerprint two-dimensional barcode/two-dimensional code/Name Shengdelong Origin or Source Myanmar Fingerprint 45595655132311242311334578233656.png]

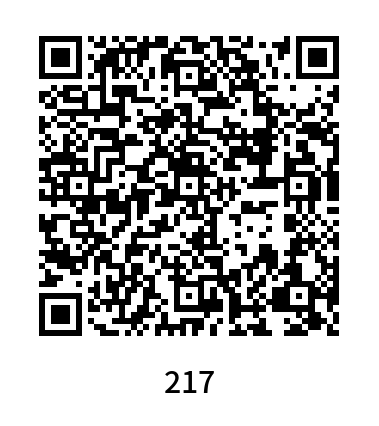

Supplement: Supplementary file 1 [file ijms-25-13625-s001.zip › Figure S1 Fingerprint two-dimensional barcode/two-dimensional code/Name Shisheng 1 Origin or Source China Fingerprint 25461118562212141355235666144656.png]

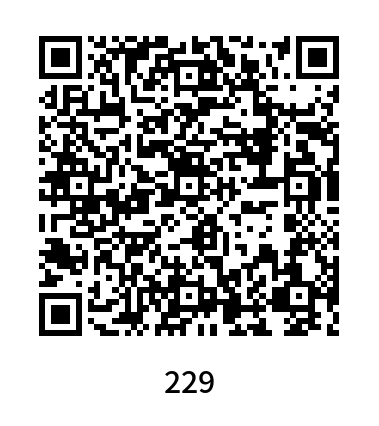

Supplement: Supplementary file 1 [file ijms-25-13625-s001.zip › Figure S1 Fingerprint two-dimensional barcode/two-dimensional code/Name Shisheng 2 Origin or Source China Fingerprint 25661118562612141355235666144656.png]

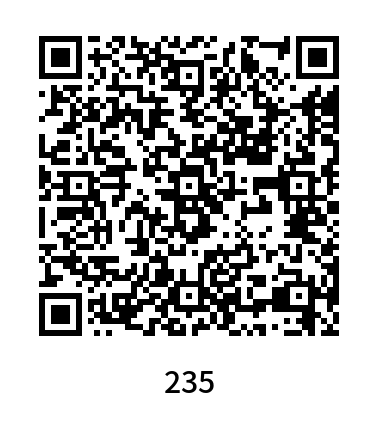

Supplement: Supplementary file 1 [file ijms-25-13625-s001.zip › Figure S1 Fingerprint two-dimensional barcode/two-dimensional code/Name Shisheng Origin or Source China Fingerprint 25461658132244342344242316264645.png]

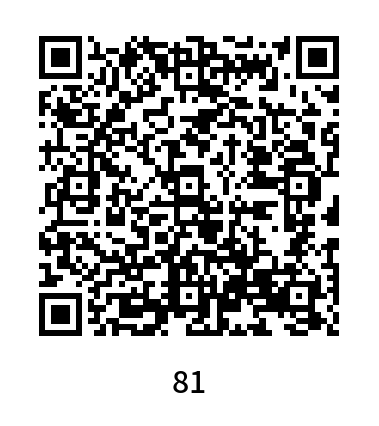

Supplement: Supplementary file 1 [file ijms-25-13625-s001.zip › Figure S1 Fingerprint two-dimensional barcode/two-dimensional code/Name Siji No. 1 Origin or Source Thailand Fingerprint 44885555564444341311343556166655.png]

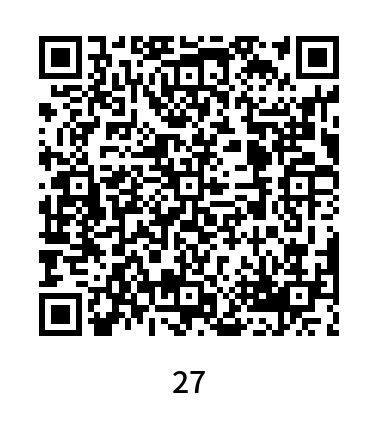

Supplement: Supplementary file 1 [file ijms-25-13625-s001.zip › Figure S1 Fingerprint two-dimensional barcode/two-dimensional code/Name Siji Origin or Source Thailand Fingerprint 45465522572325332311232313564636.png]

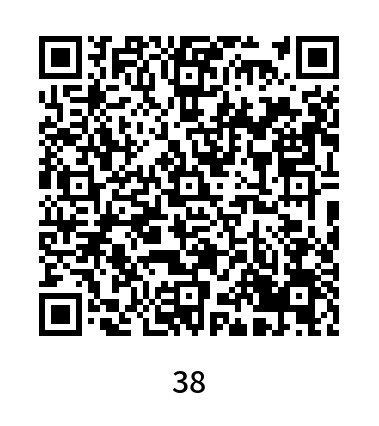

Supplement: Supplementary file 1 [file ijms-25-13625-s001.zip › Figure S1 Fingerprint two-dimensional barcode/two-dimensional code/Name Simmond Origin or Source America Fingerprint 26117718562314341444124611466655.png]

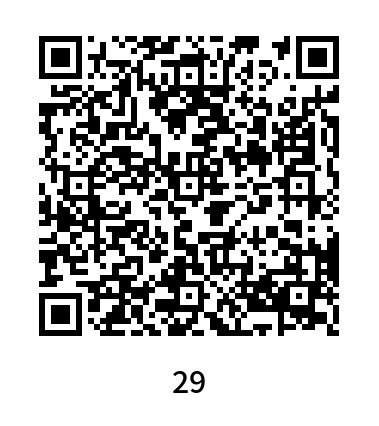

Supplement: Supplementary file 1 [file ijms-25-13625-s001.zip › Figure S1 Fingerprint two-dimensional barcode/two-dimensional code/Name Smith Origin or Source America Fingerprint 26361155352324331344342411263435.png]

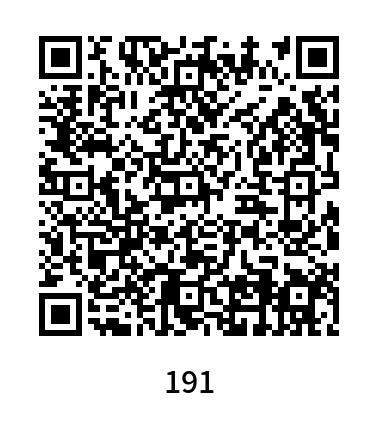

Supplement: Supplementary file 1 [file ijms-25-13625-s001.zip › Figure S1 Fingerprint two-dimensional barcode/two-dimensional code/Name Spooner Origin or Source Australia Fingerprint 46446635132414133415232515254656.png]

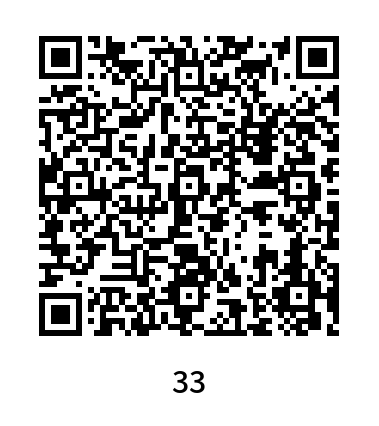

Supplement: Supplementary file 1 [file ijms-25-13625-s001.zip › Figure S1 Fingerprint two-dimensional barcode/two-dimensional code/Name Springfels Origin or Source America Fingerprint 25661555133415143444242611245656.png]

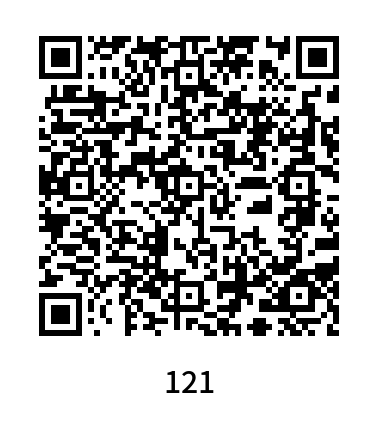

Supplement: Supplementary file 1 [file ijms-25-13625-s001.zip › Figure S1 Fingerprint two-dimensional barcode/two-dimensional code/Name Tailand hong Origin or Source Thailand Fingerprint 24441677113414132315235635134636.png]

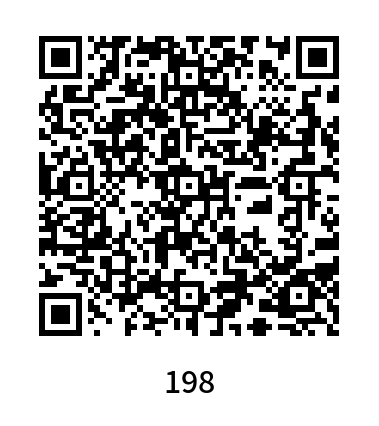

Supplement: Supplementary file 1 [file ijms-25-13625-s001.zip › Figure S1 Fingerprint two-dimensional barcode/two-dimensional code/Name Tailand mang Origin or Source Thailand Fingerprint 24351415132222133515243568266634.png]

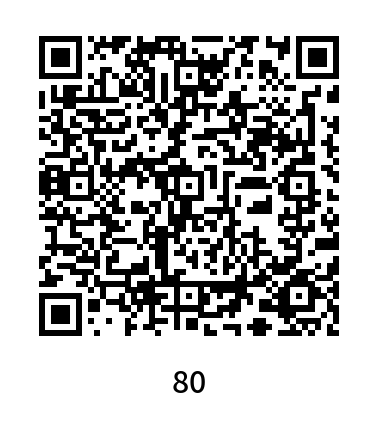

Supplement: Supplementary file 1 [file ijms-25-13625-s001.zip › Figure S1 Fingerprint two-dimensional barcode/two-dimensional code/Name Tailand No.2 Origin or Source Thailand Fingerprint 23455677151325331355453518113434.png]

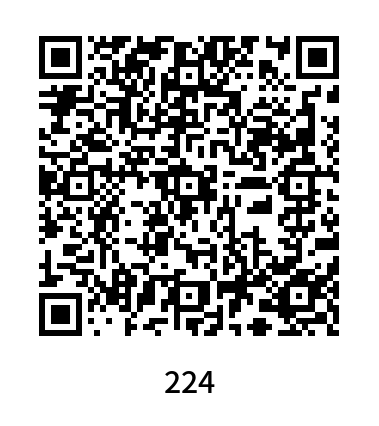

Supplement: Supplementary file 1 [file ijms-25-13625-s001.zip › Figure S1 Fingerprint two-dimensional barcode/two-dimensional code/Name Tailand qing Origin or Source Thailand Fingerprint 23465688152325331355353518113434.png]

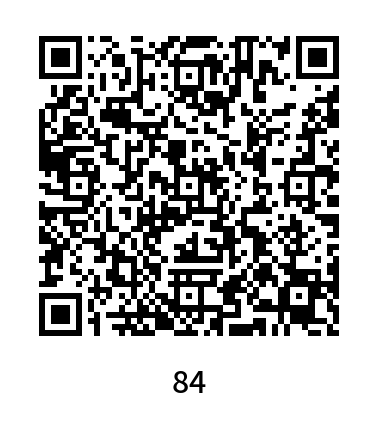

Supplement: Supplementary file 1 [file ijms-25-13625-s001.zip › Figure S1 Fingerprint two-dimensional barcode/two-dimensional code/Name Tailand yesheng Origin or Source Thailand Fingerprint 334A6639351213242415231616124433.png]

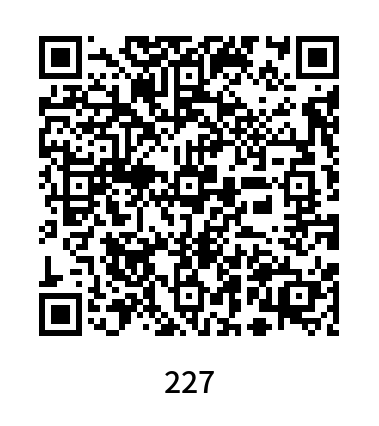

Supplement: Supplementary file 1 [file ijms-25-13625-s001.zip › Figure S1 Fingerprint two-dimensional barcode/two-dimensional code/Name Tainong No.2 Origin or Source ChinaTaiwan Fingerprint 23445615162424133414242416224535.png]

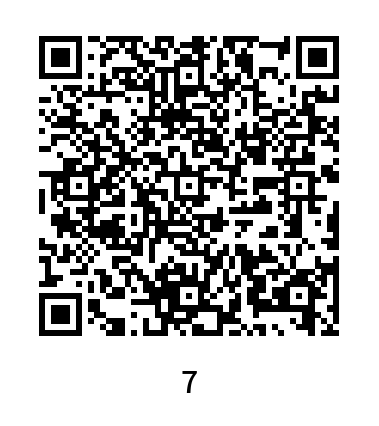

Supplement: Supplementary file 1 [file ijms-25-13625-s001.zip › Figure S1 Fingerprint two-dimensional barcode/two-dimensional code/Name Tainong1 Origin or Source ChinaTaiwan Fingerprint 34451155161424133444254558124656.png]

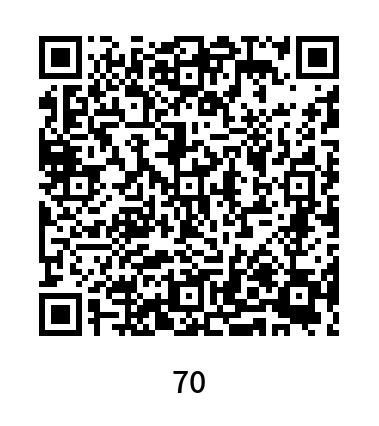

Supplement: Supplementary file 1 [file ijms-25-13625-s001.zip › Figure S1 Fingerprint two-dimensional barcode/two-dimensional code/Name Thailand Carabo Origin or Source Thailand Fingerprint 24451158131325112355455656113436.png]
