# Supplementary figures and images for: Genetic Diversity and Fingerprinting of 231 Mango Germplasm Using Genome SSR Markers (part 1 of 2)
Source: Int J Mol Sci. 2024 Dec 19;25(24):13625. doi: 10.3390/ijms252413625 (PMC11728225; doi:10.3390/ijms252413625)

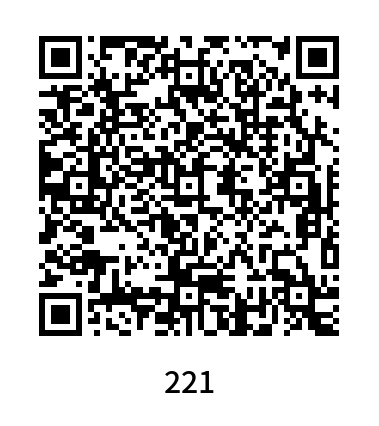

Supplement: Supplementary file 1 [file ijms-25-13625-s001.zip › Figure S1 Fingerprint two-dimensional barcode/two-dimensional code/Name 060123 Origin or Source China Fingerprint 36561155113344133444242216224456.png]

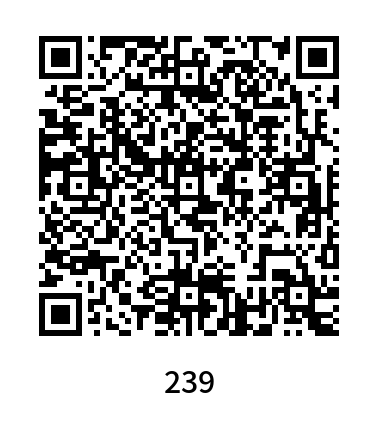

Supplement: Supplementary file 1 [file ijms-25-13625-s001.zip › Figure S1 Fingerprint two-dimensional barcode/two-dimensional code/Name 060429 Origin or Source China Fingerprint 23461615352244333313244518243636.png]

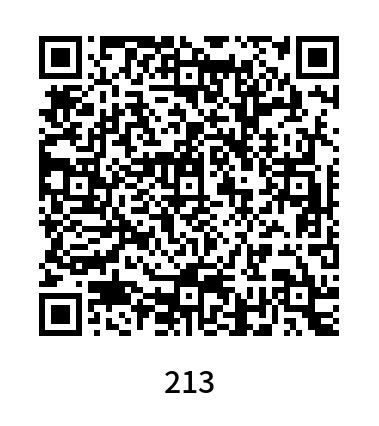

Supplement: Supplementary file 1 [file ijms-25-13625-s001.zip › Figure S1 Fingerprint two-dimensional barcode/two-dimensional code/Name 060503 Origin or Source China Fingerprint 26464615131212343315235511563656.png]

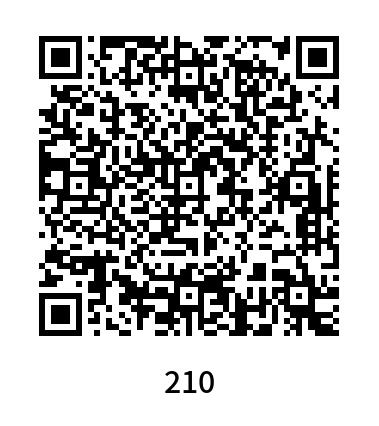

Supplement: Supplementary file 1 [file ijms-25-13625-s001.zip › Figure S1 Fingerprint two-dimensional barcode/two-dimensional code/Name 060620 Origin or Source China Fingerprint 25340055132414133414123411243455.png]

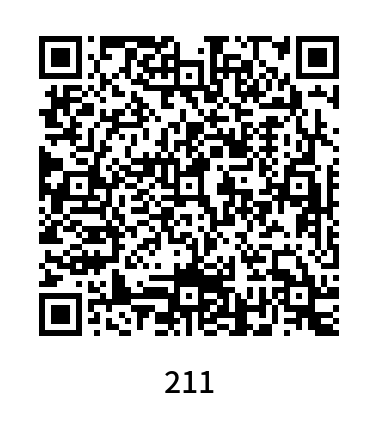

Supplement: Supplementary file 1 [file ijms-25-13625-s001.zip › Figure S1 Fingerprint two-dimensional barcode/two-dimensional code/Name 063227 Origin or Source China Fingerprint 33451115162322133444252568224655.png]

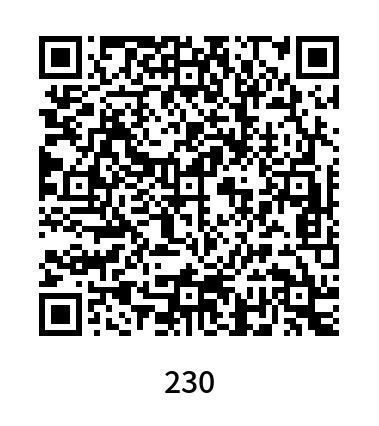

Supplement: Supplementary file 1 [file ijms-25-13625-s001.zip › Figure S1 Fingerprint two-dimensional barcode/two-dimensional code/Name 063626 Origin or Source China Fingerprint 24466655112411131344232411565656.png]

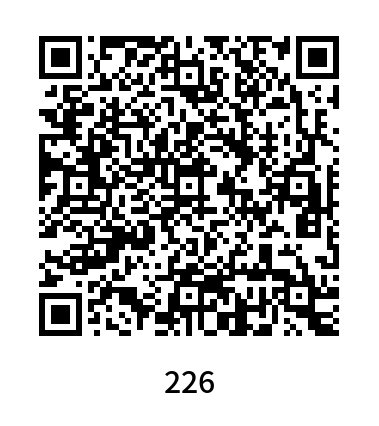

Supplement: Supplementary file 1 [file ijms-25-13625-s001.zip › Figure S1 Fingerprint two-dimensional barcode/two-dimensional code/Name 063915 Origin or Source China Fingerprint 23481118562425331355245616226656.png]

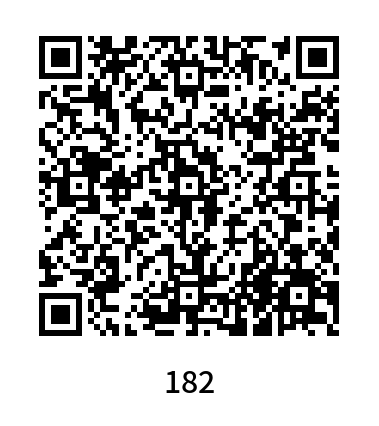

Supplement: Supplementary file 1 [file ijms-25-13625-s001.zip › Figure S1 Fingerprint two-dimensional barcode/two-dimensional code/Name 101 Origin or Source Philippines Fingerprint 22464458332214441355342318253636.png]

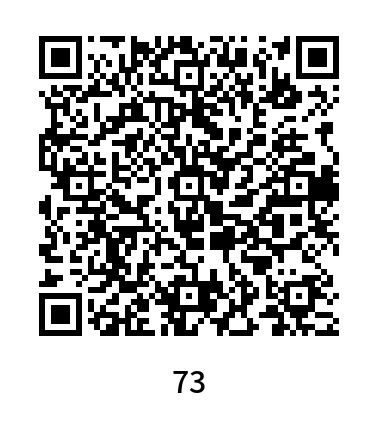

Supplement: Supplementary file 1 [file ijms-25-13625-s001.zip › Figure S1 Fingerprint two-dimensional barcode/two-dimensional code/Name 1030 Origin or Source Indonesia Fingerprint 45665555562315332415235658664636.png]

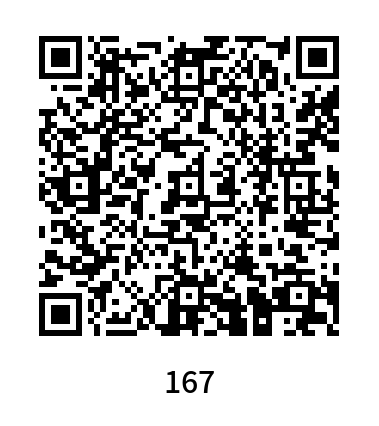

Supplement: Supplementary file 1 [file ijms-25-13625-s001.zip › Figure S1 Fingerprint two-dimensional barcode/two-dimensional code/Name 113 Origin or Source Thailand Fingerprint 24455558131325112355255656113436.png]

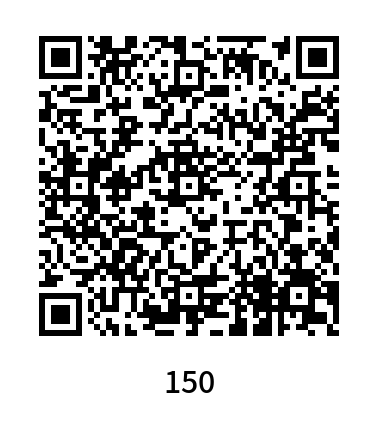

Supplement: Supplementary file 1 [file ijms-25-13625-s001.zip › Figure S1 Fingerprint two-dimensional barcode/two-dimensional code/Name 115 Origin or Source Philippines Fingerprint 22471558352225132311343656563445.png]

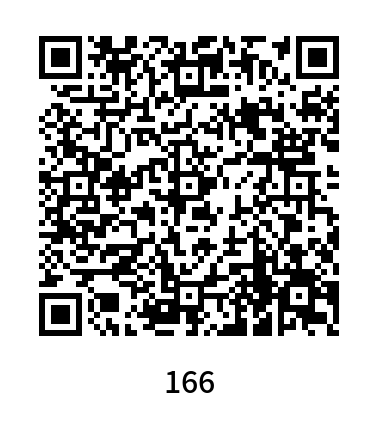

Supplement: Supplementary file 1 [file ijms-25-13625-s001.zip › Figure S1 Fingerprint two-dimensional barcode/two-dimensional code/Name 120 Origin or Source Philippines Fingerprint 22444468112345443315342568224636.png]

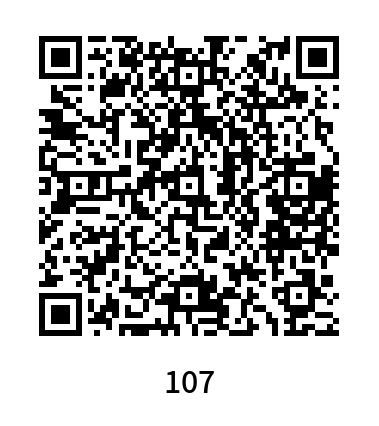

Supplement: Supplementary file 1 [file ijms-25-13625-s001.zip › Figure S1 Fingerprint two-dimensional barcode/two-dimensional code/Name 13-1 Origin or Source Israel Fingerprint 25465617152311142311345516263336.png]

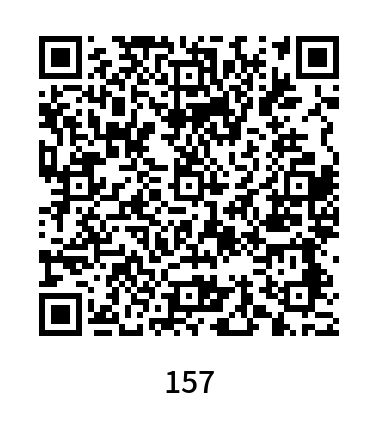

Supplement: Supplementary file 1 [file ijms-25-13625-s001.zip › Figure S1 Fingerprint two-dimensional barcode/two-dimensional code/Name 1317 Origin or Source America Fingerprint 25566615363414343434231518566666.png]

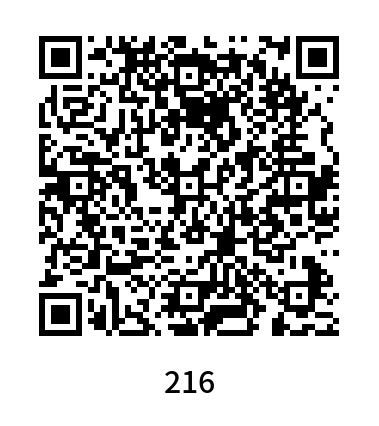

Supplement: Supplementary file 1 [file ijms-25-13625-s001.zip › Figure S1 Fingerprint two-dimensional barcode/two-dimensional code/Name 1506 Origin or Source China Fingerprint 25561118563312143355235666144656.png]

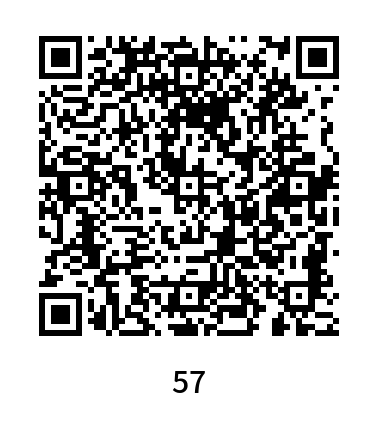

Supplement: Supplementary file 1 [file ijms-25-13625-s001.zip › Figure S1 Fingerprint two-dimensional barcode/two-dimensional code/Name 1513 Origin or Source China Fingerprint 24484655112245112311242413663645.png]

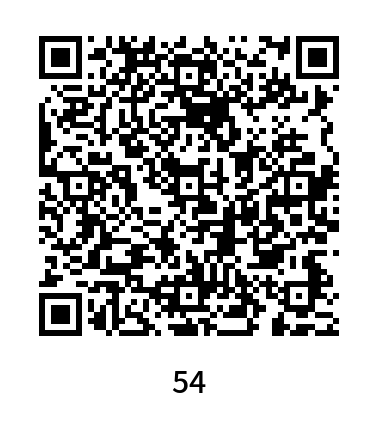

Supplement: Supplementary file 1 [file ijms-25-13625-s001.zip › Figure S1 Fingerprint two-dimensional barcode/two-dimensional code/Name 1514 Origin or Source China Fingerprint 23341168353444343444242616264655.png]

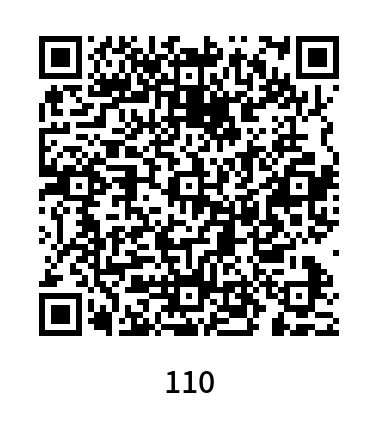

Supplement: Supplementary file 1 [file ijms-25-13625-s001.zip › Figure S1 Fingerprint two-dimensional barcode/two-dimensional code/Name 1515 Origin or Source China Fingerprint 35331515352425133444232416266635.png]

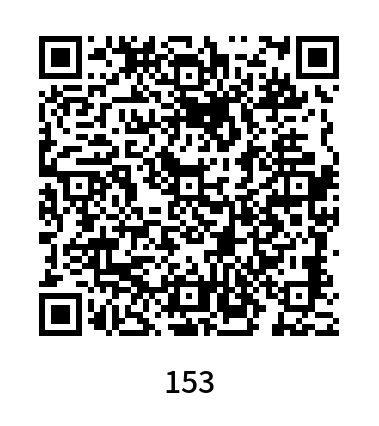

Supplement: Supplementary file 1 [file ijms-25-13625-s001.zip › Figure S1 Fingerprint two-dimensional barcode/two-dimensional code/Name 1601 Origin or Source China Fingerprint 22464458152214443355342318253636.png]

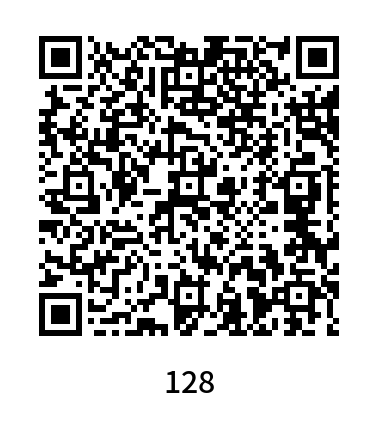

Supplement: Supplementary file 1 [file ijms-25-13625-s001.zip › Figure S1 Fingerprint two-dimensional barcode/two-dimensional code/Name 20001 Origin or Source China Fingerprint 24464628162444443315232468153413.png]

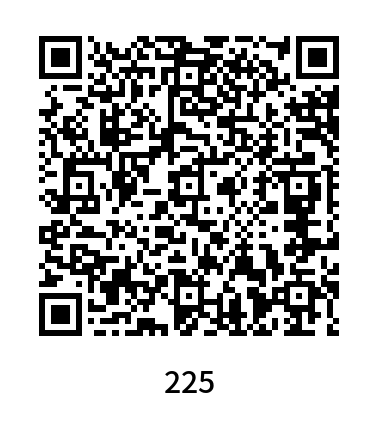

Supplement: Supplementary file 1 [file ijms-25-13625-s001.zip › Figure S1 Fingerprint two-dimensional barcode/two-dimensional code/Name 20002 Origin or Source China Fingerprint 25564515563415332415235615664646.png]

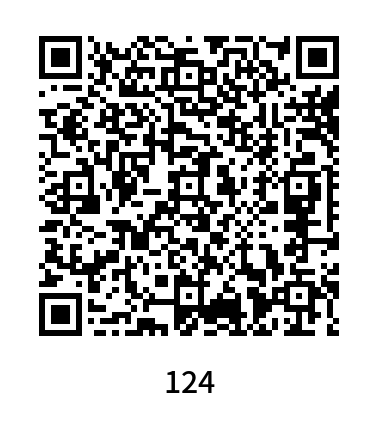

Supplement: Supplementary file 1 [file ijms-25-13625-s001.zip › Figure S1 Fingerprint two-dimensional barcode/two-dimensional code/Name 20003 Origin or Source China Fingerprint 22455557152315243515235656233433.png]

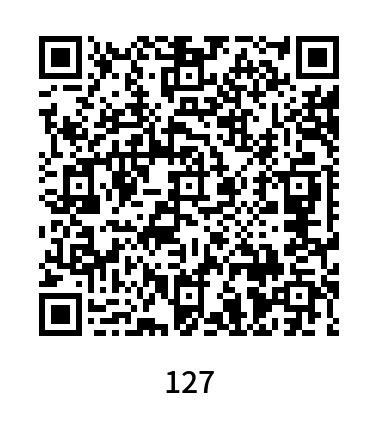

Supplement: Supplementary file 1 [file ijms-25-13625-s001.zip › Figure S1 Fingerprint two-dimensional barcode/two-dimensional code/Name 20004 Origin or Source China Fingerprint 22464625132344143515343418123413.png]

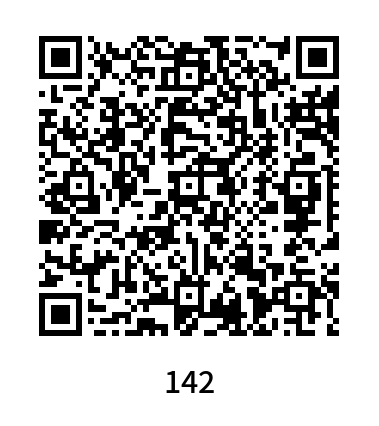

Supplement: Supplementary file 1 [file ijms-25-13625-s001.zip › Figure S1 Fingerprint two-dimensional barcode/two-dimensional code/Name 20006 Origin or Source China Fingerprint 22465658662345443555232558224636.png]

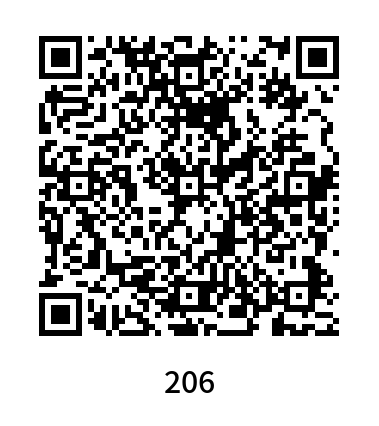

Supplement: Supplementary file 1 [file ijms-25-13625-s001.zip › Figure S1 Fingerprint two-dimensional barcode/two-dimensional code/Name 2001 Origin or Source China Fingerprint 22445558552315242315235656233433.png]

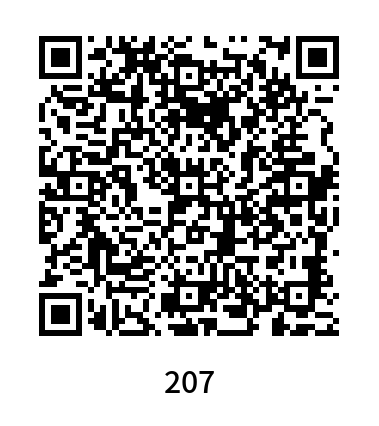

Supplement: Supplementary file 1 [file ijms-25-13625-s001.zip › Figure S1 Fingerprint two-dimensional barcode/two-dimensional code/Name 2002 Origin or Source China Fingerprint 22485558152412341355342588123346.png]

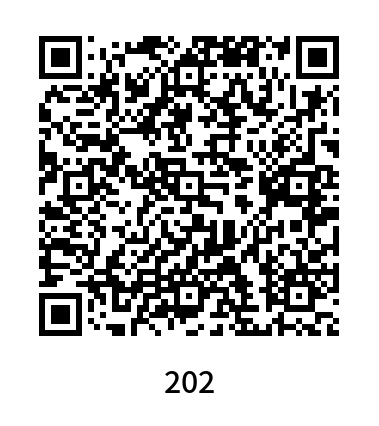

Supplement: Supplementary file 1 [file ijms-25-13625-s001.zip › Figure S1 Fingerprint two-dimensional barcode/two-dimensional code/Name 2007 Rumang Origin or Source China Fingerprint 24465655152345343544342466164636.png]

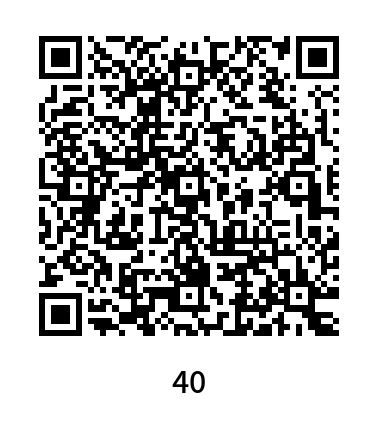

Supplement: Supplementary file 1 [file ijms-25-13625-s001.zip › Figure S1 Fingerprint two-dimensional barcode/two-dimensional code/Name 201001 Origin or Source Thailand Fingerprint 22686625362314333311344518464435.png]

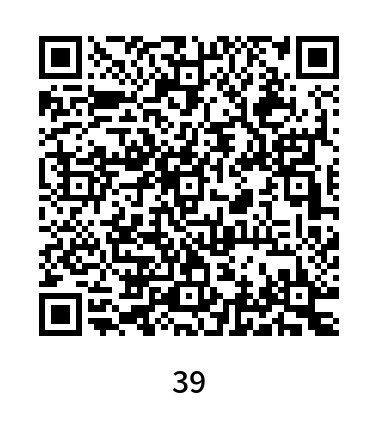

Supplement: Supplementary file 1 [file ijms-25-13625-s001.zip › Figure S1 Fingerprint two-dimensional barcode/two-dimensional code/Name 201003 Origin or Source Thailand Fingerprint 12584588672311443355236655664436.png]

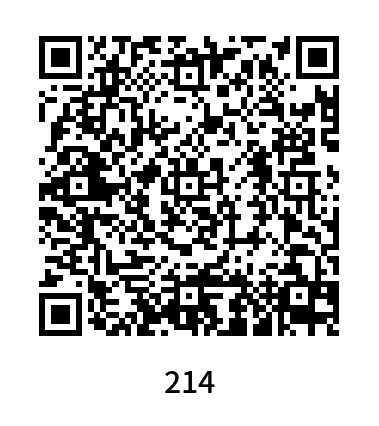

Supplement: Supplementary file 1 [file ijms-25-13625-s001.zip › Figure S1 Fingerprint two-dimensional barcode/two-dimensional code/Name 202 Origin or Source China Fingerprint 24464555162425132515122336664634.png]

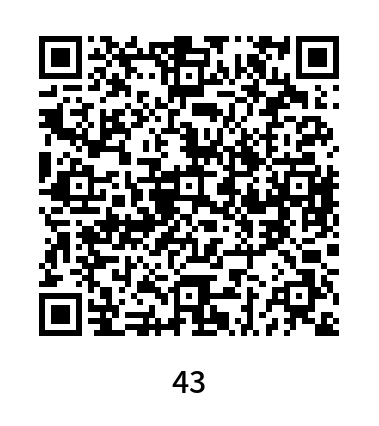

Supplement: Supplementary file 1 [file ijms-25-13625-s001.zip › Figure S1 Fingerprint two-dimensional barcode/two-dimensional code/Name 2020w Origin or Source China Fingerprint 25461168152315341344242511265545.png]

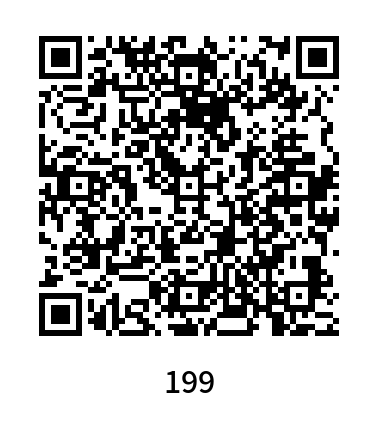

Supplement: Supplementary file 1 [file ijms-25-13625-s001.zip › Figure S1 Fingerprint two-dimensional barcode/two-dimensional code/Name 2203 Origin or Source China Fingerprint 22575557352314341344344618264636.png]

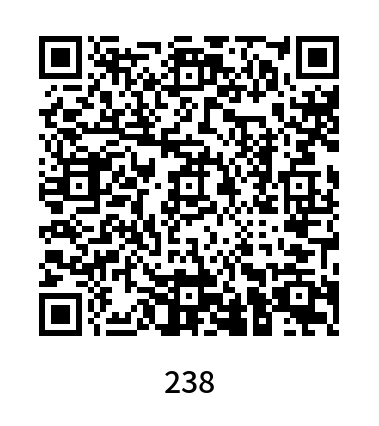

Supplement: Supplementary file 1 [file ijms-25-13625-s001.zip › Figure S1 Fingerprint two-dimensional barcode/two-dimensional code/Name 301 Origin or Source Thailand Fingerprint 25461117564612143355235666274656.png]

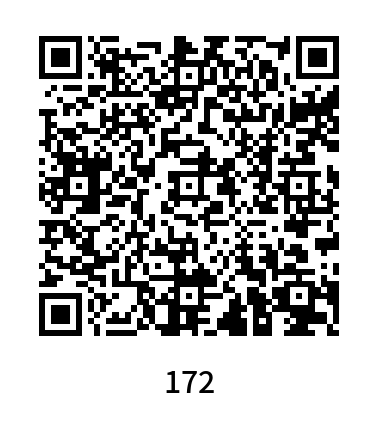

Supplement: Supplementary file 1 [file ijms-25-13625-s001.zip › Figure S1 Fingerprint two-dimensional barcode/two-dimensional code/Name 305 Origin or Source Thailand Fingerprint 24461555162215132315232656164634.png]

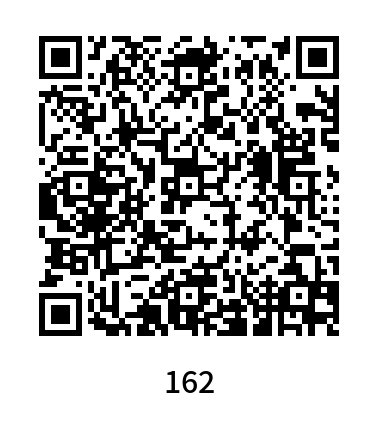

Supplement: Supplementary file 1 [file ijms-25-13625-s001.zip › Figure S1 Fingerprint two-dimensional barcode/two-dimensional code/Name 315 Origin or Source China Fingerprint 25561118153312143355235666144656.png]

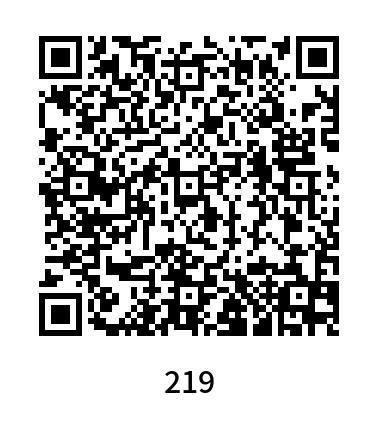

Supplement: Supplementary file 1 [file ijms-25-13625-s001.zip › Figure S1 Fingerprint two-dimensional barcode/two-dimensional code/Name 407 Origin or Source China Fingerprint 22444458352214441355342318253636.png]

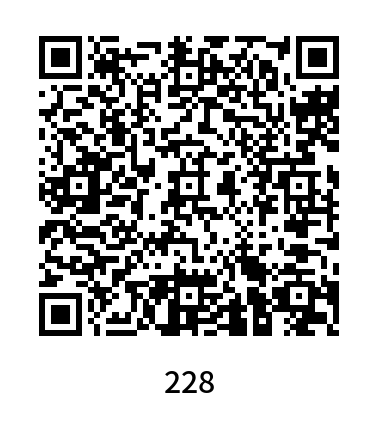

Supplement: Supplementary file 1 [file ijms-25-13625-s001.zip › Figure S1 Fingerprint two-dimensional barcode/two-dimensional code/Name 503 Origin or Source Thailand Fingerprint 23561618362414133411144516264656.png]

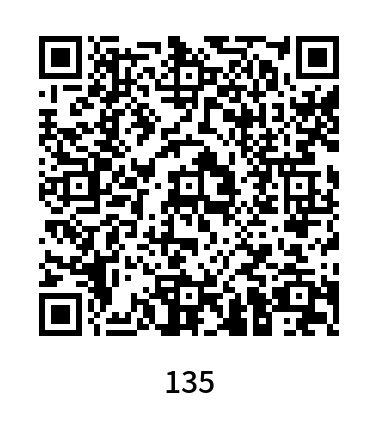

Supplement: Supplementary file 1 [file ijms-25-13625-s001.zip › Figure S1 Fingerprint two-dimensional barcode/two-dimensional code/Name 504 Origin or Source Thailand Fingerprint 24451558351325112355453656163435.png]

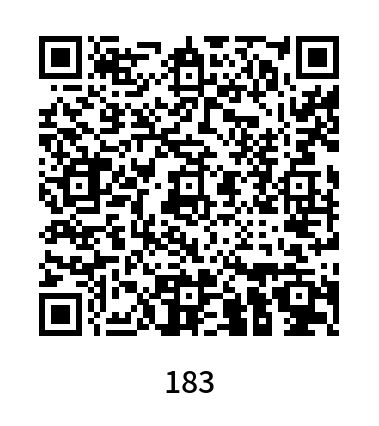

Supplement: Supplementary file 1 [file ijms-25-13625-s001.zip › Figure S1 Fingerprint two-dimensional barcode/two-dimensional code/Name 521 Origin or Source Thailand Fingerprint 22464458112214441355342318253636.png]

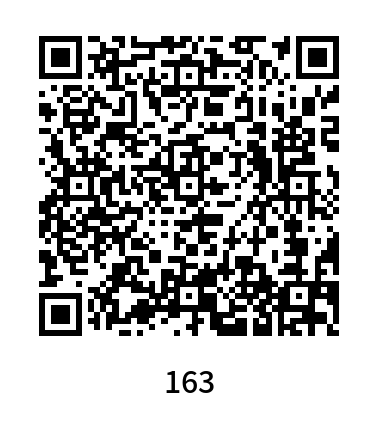

Supplement: Supplementary file 1 [file ijms-25-13625-s001.zip › Figure S1 Fingerprint two-dimensional barcode/two-dimensional code/Name 702 Origin or Source Singapore Fingerprint 22684455332444443355233516563635.png]

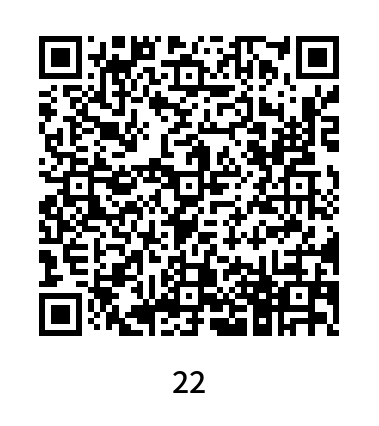

Supplement: Supplementary file 1 [file ijms-25-13625-s001.zip › Figure S1 Fingerprint two-dimensional barcode/two-dimensional code/Name 811 Origin or Source Sri Lanka Fingerprint 24454435333424143511333518253636.png]

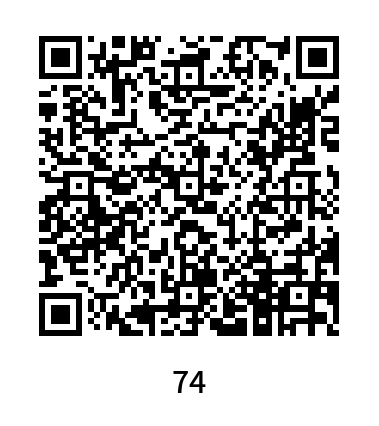

Supplement: Supplementary file 1 [file ijms-25-13625-s001.zip › Figure S1 Fingerprint two-dimensional barcode/two-dimensional code/Name 814 Origin or Source Sri Lanka Fingerprint 25586615333411112434222511265656.png]

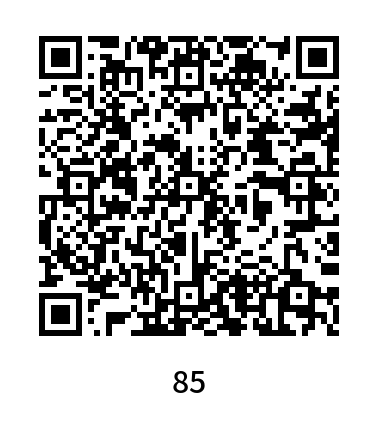

Supplement: Supplementary file 1 [file ijms-25-13625-s001.zip › Figure S1 Fingerprint two-dimensional barcode/two-dimensional code/Name Africa daxiangya Origin or Source Africa Fingerprint 23464435152444442314343368223635.png]

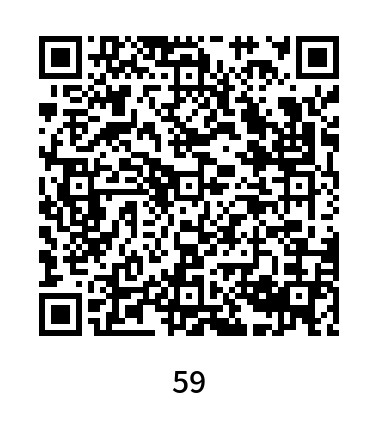

Supplement: Supplementary file 1 [file ijms-25-13625-s001.zip › Figure S1 Fingerprint two-dimensional barcode/two-dimensional code/Name Ai mang Origin or Source China Fingerprint 25455615571415132314243538663445.png]

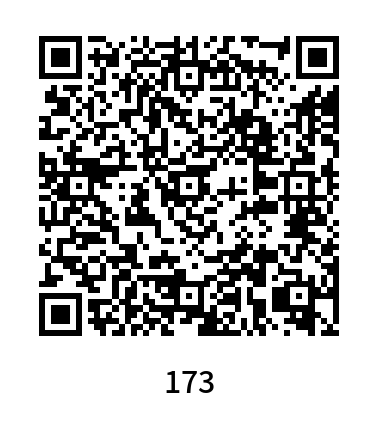

Supplement: Supplementary file 1 [file ijms-25-13625-s001.zip › Figure S1 Fingerprint two-dimensional barcode/two-dimensional code/Name Alphonso Origin or Source India Fingerprint 25561128673312141355235666144656.png]

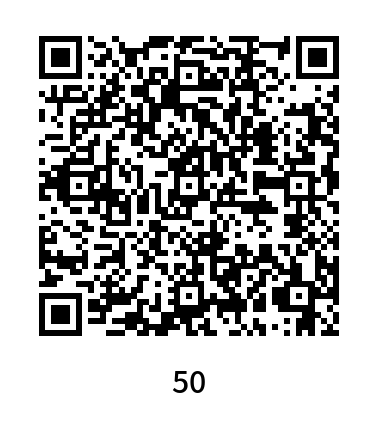

Supplement: Supplementary file 1 [file ijms-25-13625-s001.zip › Figure S1 Fingerprint two-dimensional barcode/two-dimensional code/Name Anderson Origin or Source America Fingerprint 35681611112411331444244416255655.png]

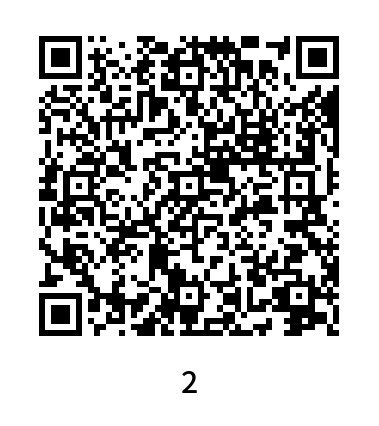

Supplement: Supplementary file 1 [file ijms-25-13625-s001.zip › Figure S1 Fingerprint two-dimensional barcode/two-dimensional code/Name Apple Origin or Source Malaysia Fingerprint 25687715364414112311232533253655.png]

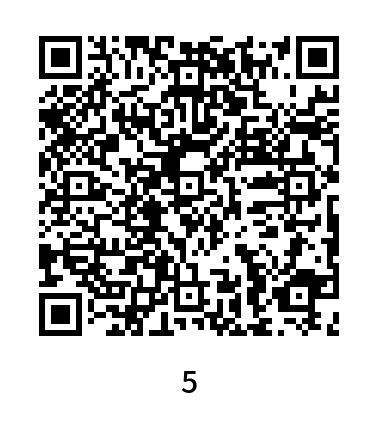

Supplement: Supplementary file 1 [file ijms-25-13625-s001.zip › Figure S1 Fingerprint two-dimensional barcode/two-dimensional code/Name Arumanis B Origin or Source Indonesia Fingerprint 25454655562644343344342336243635.png]

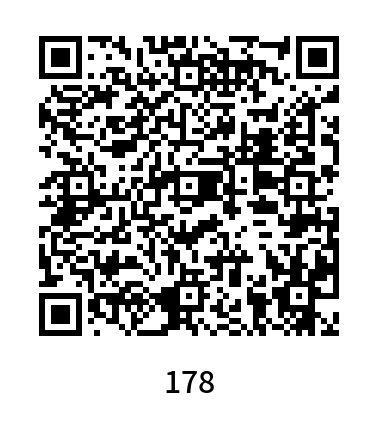

Supplement: Supplementary file 1 [file ijms-25-13625-s001.zip › Figure S1 Fingerprint two-dimensional barcode/two-dimensional code/Name Arumanis Origin or Source Indonesia Fingerprint 25364615353311341313235558564666.png]

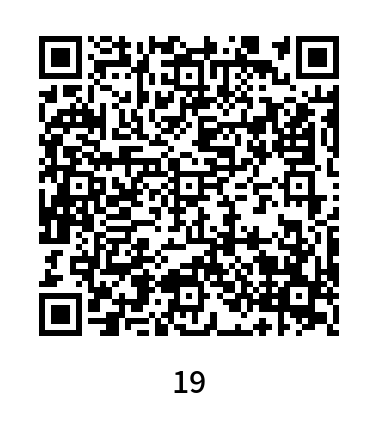

Supplement: Supplementary file 1 [file ijms-25-13625-s001.zip › Figure S1 Fingerprint two-dimensional barcode/two-dimensional code/Name Baiyu Origin or Source China Fingerprint 25464555152315231315235688663645.png]

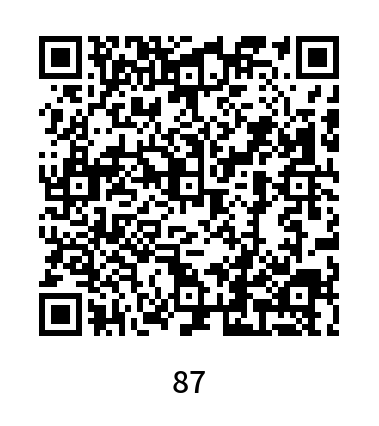

Supplement: Supplementary file 1 [file ijms-25-13625-s001.zip › Figure S1 Fingerprint two-dimensional barcode/two-dimensional code/Name Baleys Marvel Origin or Source America Fingerprint 26361615564614131311343518254636.png]

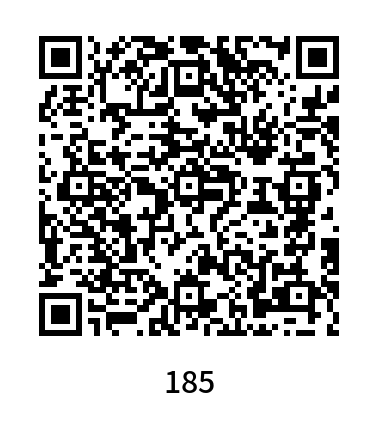

Supplement: Supplementary file 1 [file ijms-25-13625-s001.zip › Figure S1 Fingerprint two-dimensional barcode/two-dimensional code/Name Bamang Origin or Source Brazil Fingerprint 225A4458383314441355342318263636.png]

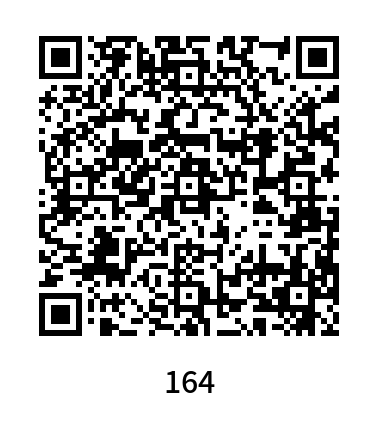

Supplement: Supplementary file 1 [file ijms-25-13625-s001.zip › Figure S1 Fingerprint two-dimensional barcode/two-dimensional code/Name Bambaroo Origin or Source Australia Fingerprint 46366635552614133415332515254656.png]

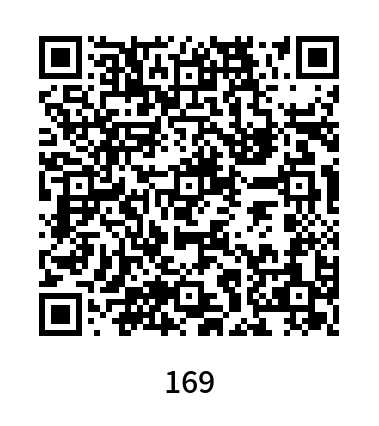

Supplement: Supplementary file 1 [file ijms-25-13625-s001.zip › Figure S1 Fingerprint two-dimensional barcode/two-dimensional code/Name Banganpali Origin or Source India Fingerprint 27564411353312131314232216443655.png]

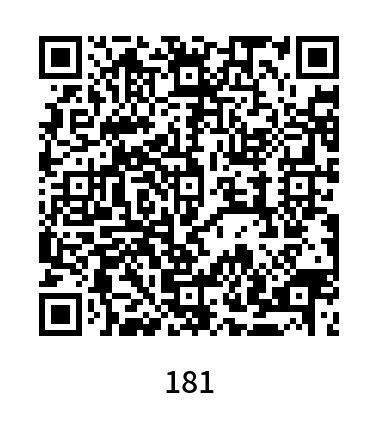

Supplement: Supplementary file 1 [file ijms-25-13625-s001.zip › Figure S1 Fingerprint two-dimensional barcode/two-dimensional code/Name Baodaohuang Origin or Source Cambodia Fingerprint 25561558362312142315235666144656.png]

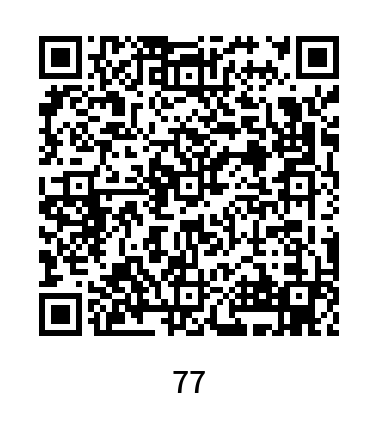

Supplement: Supplementary file 1 [file ijms-25-13625-s001.zip › Figure S1 Fingerprint two-dimensional barcode/two-dimensional code/Name Baozian Origin or Source China Fingerprint 25451115561245112355454666144656.png]

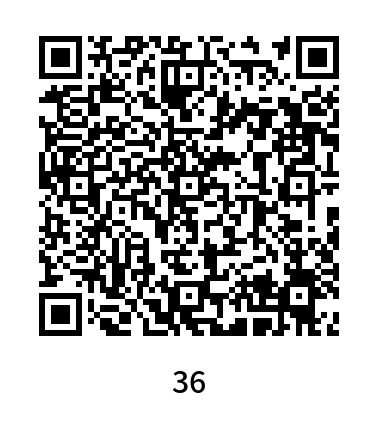

Supplement: Supplementary file 1 [file ijms-25-13625-s001.zip › Figure S1 Fingerprint two-dimensional barcode/two-dimensional code/Name Beverly Origin or Source America Fingerprint 23361111133412133314442416253415.png]

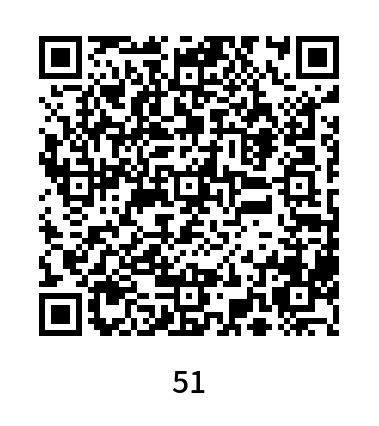

Supplement: Supplementary file 1 [file ijms-25-13625-s001.zip › Figure S1 Fingerprint two-dimensional barcode/two-dimensional code/Name Bombay green Origin or Source India Fingerprint 24356655353614143411231558556666.png]

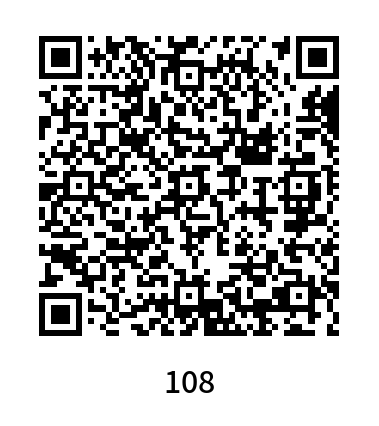

Supplement: Supplementary file 1 [file ijms-25-13625-s001.zip › Figure S1 Fingerprint two-dimensional barcode/two-dimensional code/Name Carrie Origin or Source America Fingerprint 25341155353312123414231215473656.png]

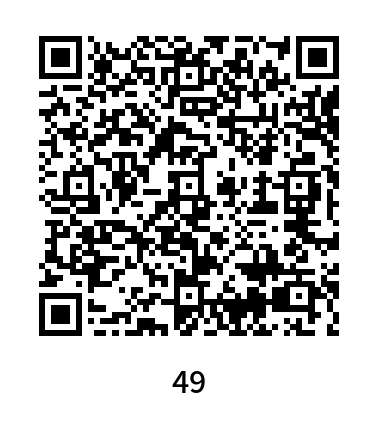

Supplement: Supplementary file 1 [file ijms-25-13625-s001.zip › Figure S1 Fingerprint two-dimensional barcode/two-dimensional code/Name Chenpi Origin or Source China Fingerprint 25681555353314343415343556666655.png]

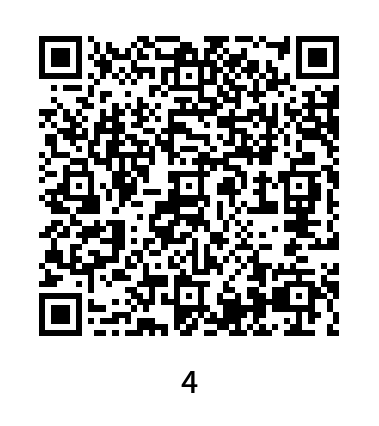

Supplement: Supplementary file 1 [file ijms-25-13625-s001.zip › Figure S1 Fingerprint two-dimensional barcode/two-dimensional code/Name Chishu Origin or Source China Fingerprint 25464558132315343435243588465666.png]

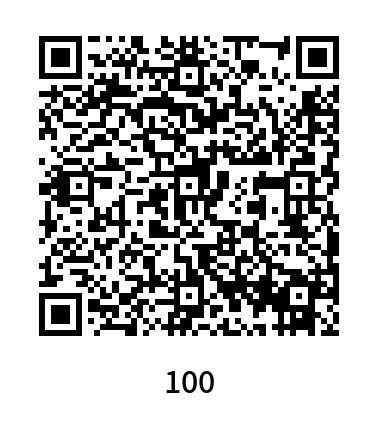

Supplement: Supplementary file 1 [file ijms-25-13625-s001.zip › Figure S1 Fingerprint two-dimensional barcode/two-dimensional code/Name Chocanon Origin or Source Thailand Fingerprint 44445525152225332511232313564436.png]

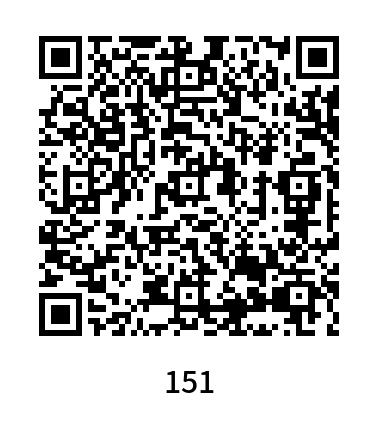

Supplement: Supplementary file 1 [file ijms-25-13625-s001.zip › Figure S1 Fingerprint two-dimensional barcode/two-dimensional code/Name Chowsa Origin or Source India Fingerprint 22445577112245343315443618264646.png]

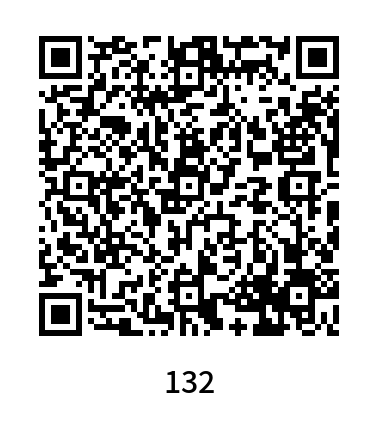

Supplement: Supplementary file 1 [file ijms-25-13625-s001.zip › Figure S1 Fingerprint two-dimensional barcode/two-dimensional code/Name Chuanmang Origin or Source China Fingerprint 25461158132315343435243588466646.png]

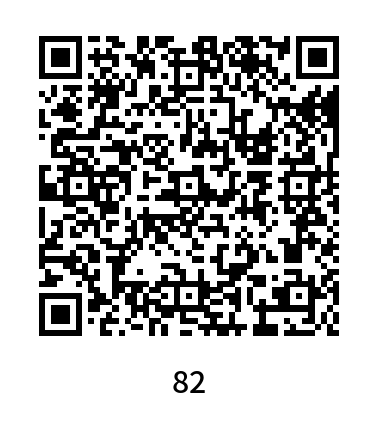

Supplement: Supplementary file 1 [file ijms-25-13625-s001.zip › Figure S1 Fingerprint two-dimensional barcode/two-dimensional code/Name Cuba No.1 Origin or Source Cuba Fingerprint 24454435353424142315333518273636.png]

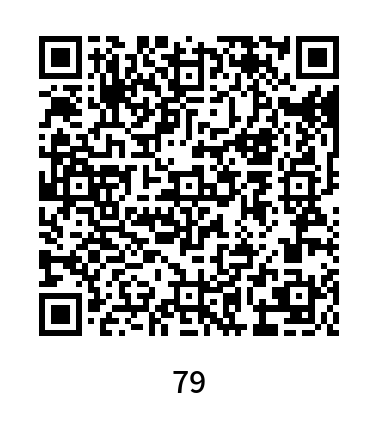

Supplement: Supplementary file 1 [file ijms-25-13625-s001.zip › Figure S1 Fingerprint two-dimensional barcode/two-dimensional code/Name Cuba No.2 Origin or Source Cuba Fingerprint 36467715152414333414243416264635.png]

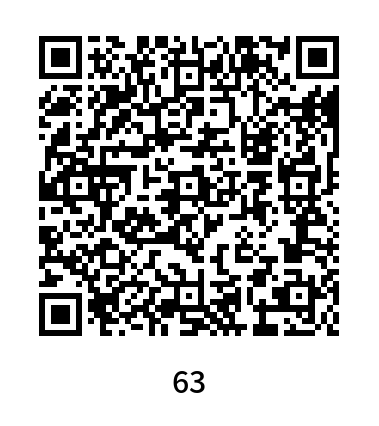

Supplement: Supplementary file 1 [file ijms-25-13625-s001.zip › Figure S1 Fingerprint two-dimensional barcode/two-dimensional code/Name Cuba No.3 Origin or Source Cuba Fingerprint 34674622562334121225244536123433.png]

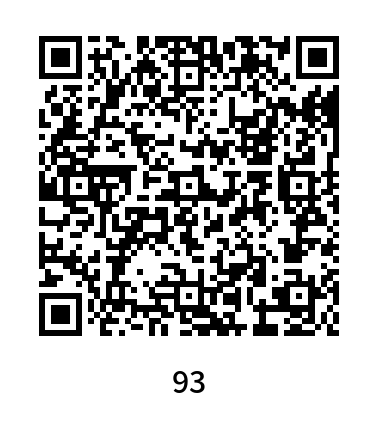

Supplement: Supplementary file 1 [file ijms-25-13625-s001.zip › Figure S1 Fingerprint two-dimensional barcode/two-dimensional code/Name Cuba No.4 Origin or Source Cuba Fingerprint 22464468132214441345342318253636.png]

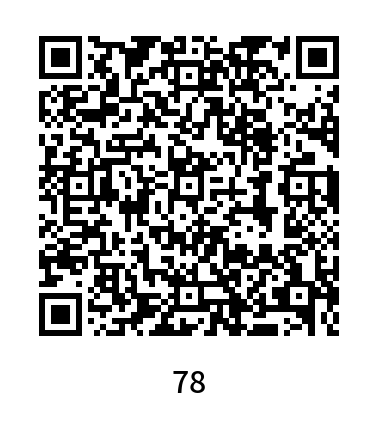

Supplement: Supplementary file 1 [file ijms-25-13625-s001.zip › Figure S1 Fingerprint two-dimensional barcode/two-dimensional code/Name Cubasankeli Origin or Source Cuba Fingerprint 25466655112412232444234511443435.png]

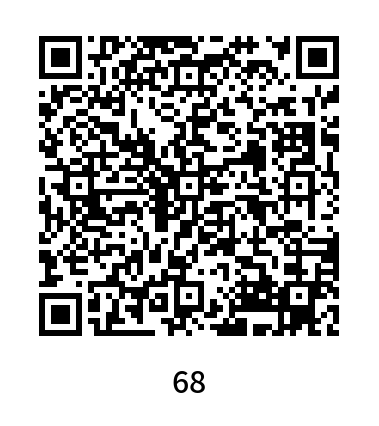

Supplement: Supplementary file 1 [file ijms-25-13625-s001.zip › Figure S1 Fingerprint two-dimensional barcode/two-dimensional code/Name Dabaiyu Origin or Source China Fingerprint 23455588562355232355453558164424.png]

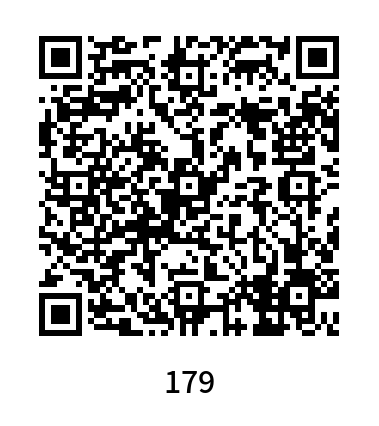

Supplement: Supplementary file 1 [file ijms-25-13625-s001.zip › Figure S1 Fingerprint two-dimensional barcode/two-dimensional code/Name Dasannian Origin or Source China Fingerprint 25461158132315331315245618264656.png]

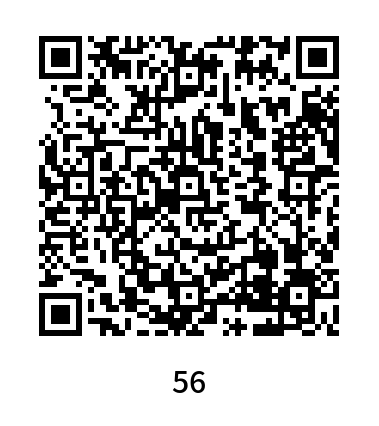

Supplement: Supplementary file 1 [file ijms-25-13625-s001.zip › Figure S1 Fingerprint two-dimensional barcode/two-dimensional code/Name Dashehari Origin or Source India Fingerprint 24686655563444343415333566163655.png]

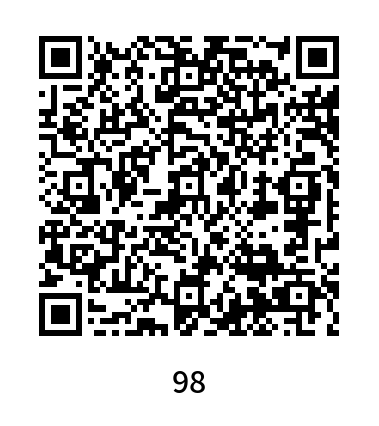

Supplement: Supplementary file 1 [file ijms-25-13625-s001.zip › Figure S1 Fingerprint two-dimensional barcode/two-dimensional code/Name Dazhou Origin or Source China Fingerprint 22464488352345242315342658354636.png]

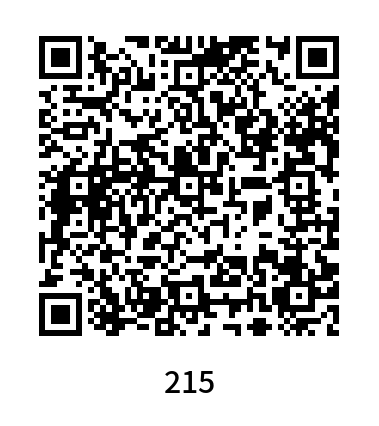

Supplement: Supplementary file 1 [file ijms-25-13625-s001.zip › Figure S1 Fingerprint two-dimensional barcode/two-dimensional code/Name Dongzhenhong Origin or Source China Fingerprint 45561558351215123344233415664535.png]

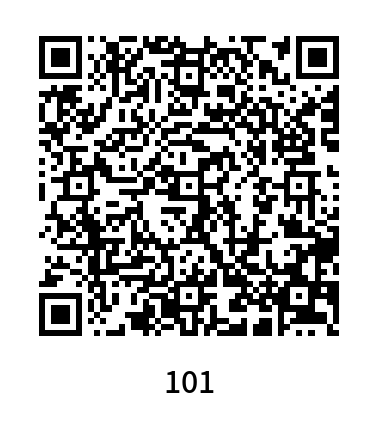

Supplement: Supplementary file 1 [file ijms-25-13625-s001.zip › Figure S1 Fingerprint two-dimensional barcode/two-dimensional code/Name Dot Origin or Source America Fingerprint 35465615353314131314122513125655.png]

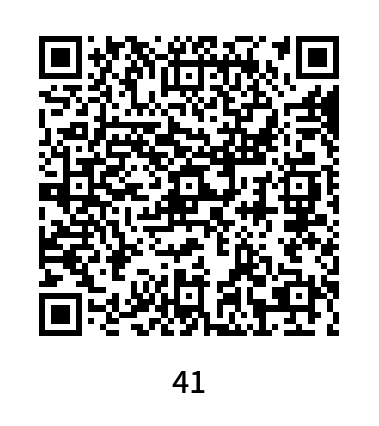

Supplement: Supplementary file 1 [file ijms-25-13625-s001.zip › Figure S1 Fingerprint two-dimensional barcode/two-dimensional code/Name Duncan Origin or Source America Fingerprint 24455557351215122415455515124436.png]

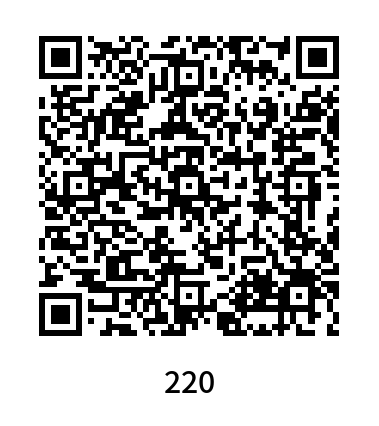

Supplement: Supplementary file 1 [file ijms-25-13625-s001.zip › Figure S1 Fingerprint two-dimensional barcode/two-dimensional code/Name Duohua Origin or Source Thailand Fingerprint 44465525562225332511232313564436.png]

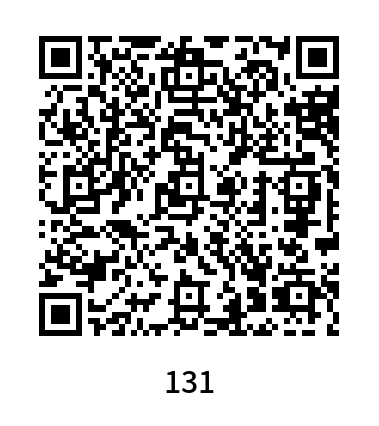

Supplement: Supplementary file 1 [file ijms-25-13625-s001.zip › Figure S1 Fingerprint two-dimensional barcode/two-dimensional code/Name Edward Origin or Source India Fingerprint 23461555162411232411444515222456.png]

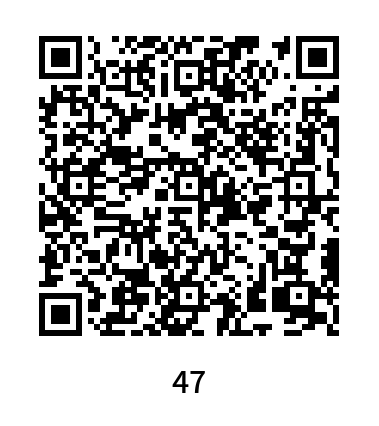

Supplement: Supplementary file 1 [file ijms-25-13625-s001.zip › Figure S1 Fingerprint two-dimensional barcode/two-dimensional code/Name Eldon Origin or Source America Fingerprint 247A1468562414122315333516566655.png]

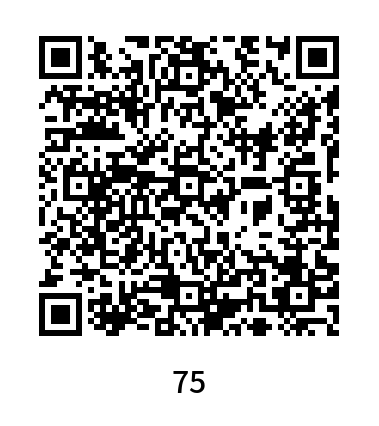

Supplement: Supplementary file 1 [file ijms-25-13625-s001.zip › Figure S1 Fingerprint two-dimensional barcode/two-dimensional code/Name Fengshunwuhe Origin or Source China Fingerprint 24445558162255232315343658664434.png]

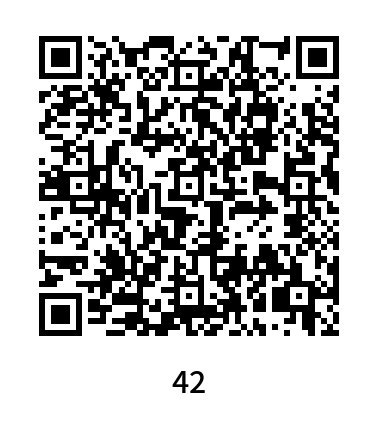

Supplement: Supplementary file 1 [file ijms-25-13625-s001.zip › Figure S1 Fingerprint two-dimensional barcode/two-dimensional code/Name Florigon Origin or Source America Fingerprint 56341115561312143411341411673636.png]

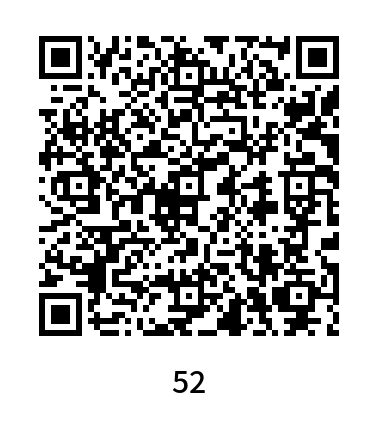

Supplement: Supplementary file 1 [file ijms-25-13625-s001.zip › Figure S1 Fingerprint two-dimensional barcode/two-dimensional code/Name Ford Origin or Source America Fingerprint 35681611562344331344122316223655.png]

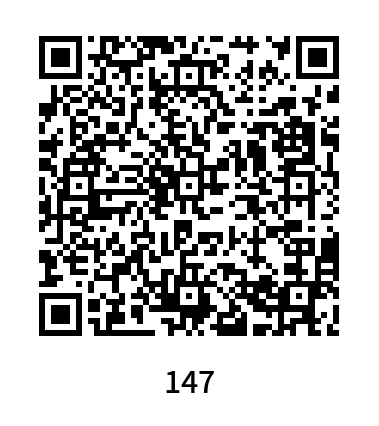

Supplement: Supplementary file 1 [file ijms-25-13625-s001.zip › Figure S1 Fingerprint two-dimensional barcode/two-dimensional code/Name Fu No.1 Origin or Source China Fingerprint 55686655333414134413232511246655.png]

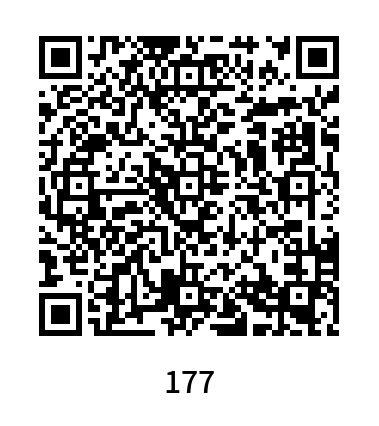

Supplement: Supplementary file 1 [file ijms-25-13625-s001.zip › Figure S1 Fingerprint two-dimensional barcode/two-dimensional code/Name Fu No.2 Origin or Source China Fingerprint 25561117553312143355234516144656.png]

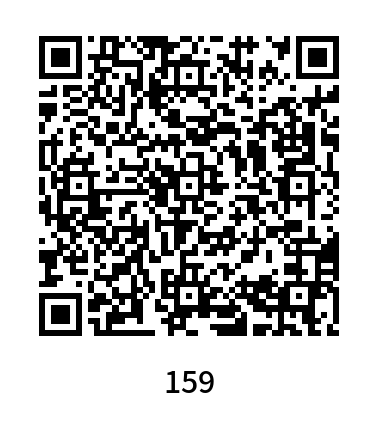

Supplement: Supplementary file 1 [file ijms-25-13625-s001.zip › Figure S1 Fingerprint two-dimensional barcode/two-dimensional code/Name Fu No.3 Origin or Source China Fingerprint 25661825113314133315245568144636.png]

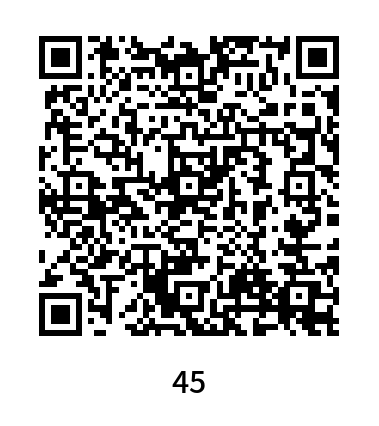

Supplement: Supplementary file 1 [file ijms-25-13625-s001.zip › Figure S1 Fingerprint two-dimensional barcode/two-dimensional code/Name Gangguoshishengzhong Origin or Source Congo Fingerprint 23484413553412343414232311223635.png]

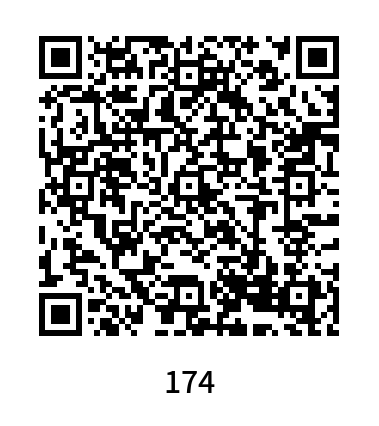

Supplement: Supplementary file 1 [file ijms-25-13625-s001.zip › Figure S1 Fingerprint two-dimensional barcode/two-dimensional code/Name Ganhong Origin or Source ChinaTaiwan Fingerprint 34361155352214132344232411264655.png]

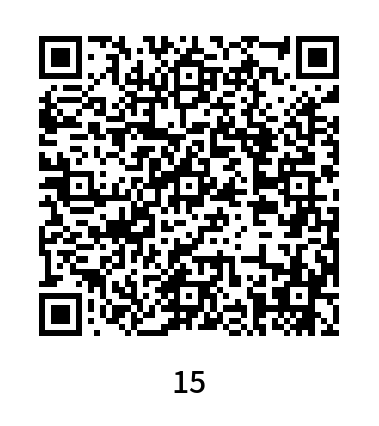

Supplement: Supplementary file 1 [file ijms-25-13625-s001.zip › Figure S1 Fingerprint two-dimensional barcode/two-dimensional code/Name GeDong R Origin or Source Indonesia Fingerprint 27466618332345343544233468463656.png]

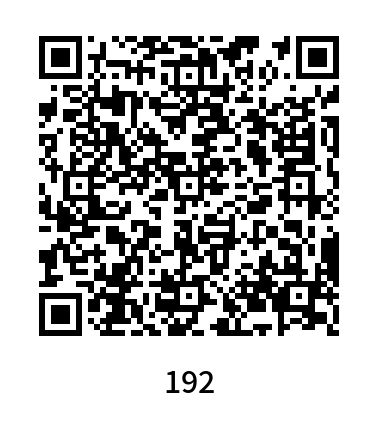

Supplement: Supplementary file 1 [file ijms-25-13625-s001.zip › Figure S1 Fingerprint two-dimensional barcode/two-dimensional code/Name Glenn Origin or Source America Fingerprint 23681617134414133411344516264656.png]

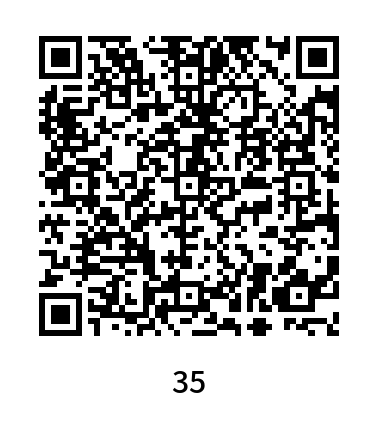

Supplement: Supplementary file 1 [file ijms-25-13625-s001.zip › Figure S1 Fingerprint two-dimensional barcode/two-dimensional code/Name Gold Lippens Origin or Source America Fingerprint 56661155352415112314242238255635.png]

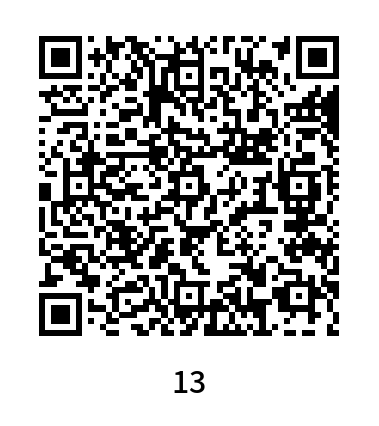

Supplement: Supplementary file 1 [file ijms-25-13625-s001.zip › Figure S1 Fingerprint two-dimensional barcode/two-dimensional code/Name Graham Origin or Source America Fingerprint 47354655133345341414231215453656.png]

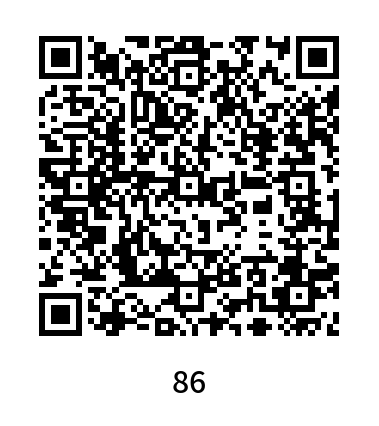

Supplement: Supplementary file 1 [file ijms-25-13625-s001.zip › Figure S1 Fingerprint two-dimensional barcode/two-dimensional code/Name Guangxi No.4 Origin or Source China Fingerprint 34464558132445331411122266164635.png]

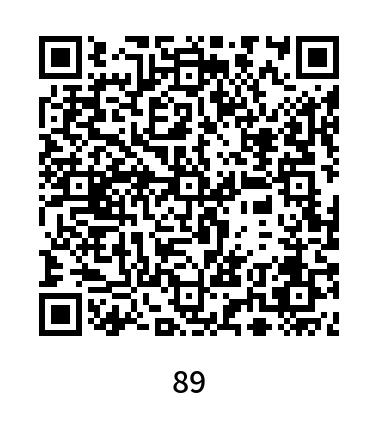

Supplement: Supplementary file 1 [file ijms-25-13625-s001.zip › Figure S1 Fingerprint two-dimensional barcode/two-dimensional code/Name Guangxi No.8 Origin or Source China Fingerprint 27665655352414331315345513343455.png]

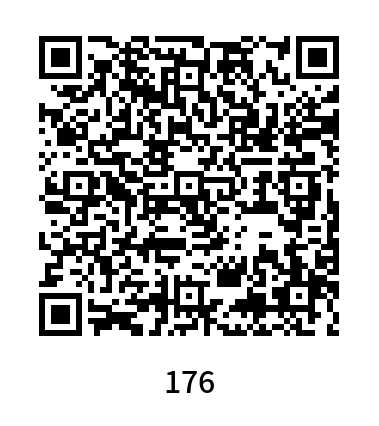

Supplement: Supplementary file 1 [file ijms-25-13625-s001.zip › Figure S1 Fingerprint two-dimensional barcode/two-dimensional code/Name Guifei Origin or Source ChinaTaiwan Fingerprint 34461558382215122344343418663435.png]

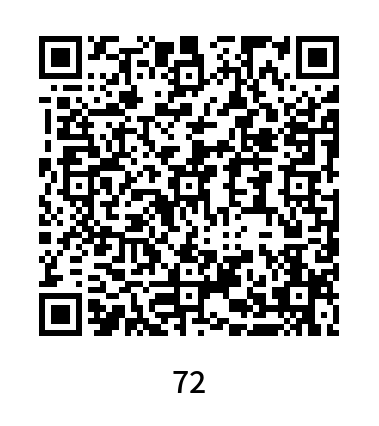

Supplement: Supplementary file 1 [file ijms-25-13625-s001.zip › Figure S1 Fingerprint two-dimensional barcode/two-dimensional code/Name Guinea No.2 Origin or Source Guinea Fingerprint 67364415563615331344222316463355.png]

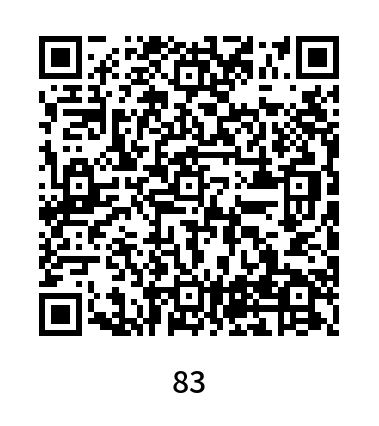

Supplement: Supplementary file 1 [file ijms-25-13625-s001.zip › Figure S1 Fingerprint two-dimensional barcode/two-dimensional code/Name Guinea No1 Origin or Source Guinea Fingerprint 26467715352424133415244568253413.png]

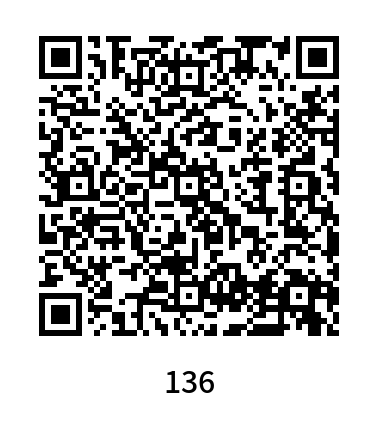

Supplement: Supplementary file 1 [file ijms-25-13625-s001.zip › Figure S1 Fingerprint two-dimensional barcode/two-dimensional code/Name Guire No.10 Origin or Source China Fingerprint 22464458152214443355342318253636.png]

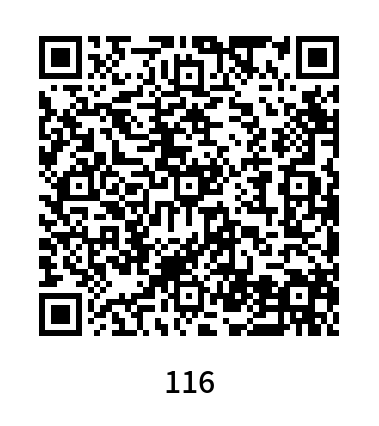

Supplement: Supplementary file 1 [file ijms-25-13625-s001.zip › Figure S1 Fingerprint two-dimensional barcode/two-dimensional code/Name Guire No.82 Origin or Source China Fingerprint 45464555462315332333232558564645.png]

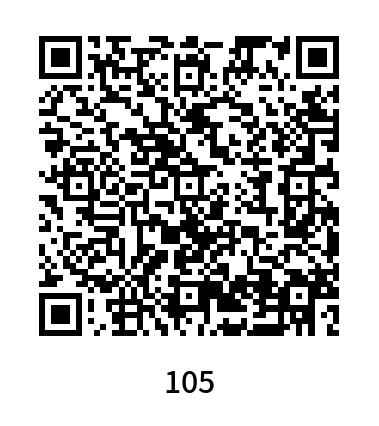

Supplement: Supplementary file 1 [file ijms-25-13625-s001.zip › Figure S1 Fingerprint two-dimensional barcode/two-dimensional code/Name Guirewuming Origin or Source China Fingerprint 45464535132315332315232515464635.png]

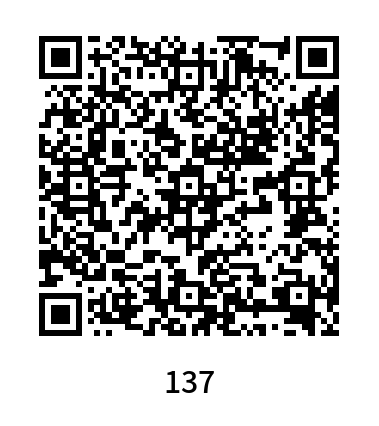

Supplement: Supplementary file 1 [file ijms-25-13625-s001.zip › Figure S1 Fingerprint two-dimensional barcode/two-dimensional code/Name Guixiang Origin or Source China Fingerprint 25664415112314343435243568265635.png]
