# Supplementary material for: Genetic Diversity and Fingerprinting of 231 Mango Germplasm Using Genome SSR Markers
Source: Int J Mol Sci. 2024 Dec 19;25(24):13625. doi: 10.3390/ijms252413625 (PMC11728225; doi:10.3390/ijms252413625)
Supplement: Supplementary file 1 [file ijms-25-13625-s001.zip › Table S5.pdf]

Table S5. Fingerprint of 229 mango accessions

No.	Name	Origin or Source	Fingerprint
1	Irwin	America	35667755562411133444242411264555
2	Apple	Malaysia	25687715364414112311232533253655
3	NamDocMai	Thailand	24444555161225231315345615164445
4	Chishu	China	25464558132315343435243588465666
5	Arumanis B	Indonesia	25454655562644343344342336243635
6	JinHuang	ChinaTaiwan	45341555132445232355334615264635
7	Tainong1	ChinaTaiwan	34451155161424133444254558124656
8	kyo savoy	Thailand	25681118113355333434222518465646
9	Renong1	Australia	36661613132212113444232215256656
10	Kent	America	35347715152424332315344518226635
11	KenSington	Australia	46366635152614133415232515256656
12	Gundoo	Indonesia	33347715111224132444122568226655
13	Graham	America	47354655133345341414231215453656
14	Wuyichang	China	45664555562315332335232558664645
15	GeDong R	Indonesia	27466618332345343544233468463656
16	Indonesia No.1	Indonesia	25681617162415133444120016455645
17	Sandersha	America	56681615364414332314242411563455
18	Indonesia No.2	Indonesia	36467715132414333414243416264635
19	Baiyu	China	25464555152315231315235688663645
20	Keitt	America	35461155262324332444244511254615
21	Peach	America	26457715112414133414341417254656
22	811	Sri Lanka	24454435333424143511333518253636
23	NamDocMai4	Thailand	25461168552315331315245618266656
24	Zillate	America	25681655352424133414242511564655
25	Mylepania	Australia	46364635352314131415234515456656
26	Webber	America	46341615573414131311343468666656
27	Siji	Thailand	45465522572325332311232313564636
28	Ruby	America	35681111354414333444122411263655
29	Smith	America	26361155352324331344342411263435
30	Julie	the West Indies	57361455362315231414122213443635
31	Tom EJ/C	Israel	55564615353314133414122518465666
32	Lippens	America	56461155152414133344242318254556
33	Springfels	America	25661555133415143444242611245656

34	Indonesia No.3	Indonesia	22455658152444141344344468164656
35	Gold Lippens	America	56661155352415112314242238255635
36	Beverly	America	23361111133412133314442416253415
37	Sensation	America	35361115562424133444242316224655
38	Simmond	America	26117718562314341444124611466655
39	201003	Thailand	12584588672311443355236655664436
40	201001	Thailand	22686625362314333311344518464435
41	Duncan	America	24455557351215122415455515124436
42	Florigon	America	56341115561312143411341411673636
43	2020w	China	25461168152315341344242511265545
45	Gangguoshishengzhong	Congo	23484413553412343414232311223635
46	Palmer	America	35347715562324132414443516226635
47	Eldon	America	247A1468562414122315333516566655
48	Nam Dok Mai Sitong	Thailand	25461168152315333511245618266656
49	Chenpi	China	25681555353314343415343556666655
50	Anderson	America	35681611112411331444244416255655
51	Bombay green	India	24356655353614143411231558556666
52	Ford	America	35681611562344331344122316223655
54	1514	China	23341168353444343444242616264655
55	Xiangjiao	Myanmar	22445588561245341315343618264646
56	Dashehari	India	24686655563444343415333566163655
57	1513	China	24484655112245112311242413663645
58	Panyu No.2	China	22465555152445332311235656664645
59	Ai mang	China	25455615571415132314243538663445
60	Shengdelong	Myanmar	45595655132311242311334578233656
61	Jianmang	Cambodia	24455558131215141315335516163445
62	Shanlin	ChinaTaiwan	35661611564414131314244411254555
63	Cuba No.3	Cuba	34674622562334121225244536123433
64	Xianluo	China	255A1155133414233415244536346635
65	Saber	America	25463438562414132314233519251335
66	kwini	Indonesia	33361115354414133444243411224555
67	Hongmangguo	America	35361115562424133444242316224635
68	Dabaiyu	China	23455588562355232355453558164424
69	Rjs000l	China	22456628571255243415454516253334
70	Thailand Carabo	Thailand	24451158131325112355455656113436

71	Thailand Shengshi	Thailand	24455558111215141315235516163445
72	Guinea No.2	Guinea	67364415563615331344222316463355
73	1030	Indonesia	45665555562315332415235658664636
74	814	Sri Lanka	25586615333411112434222511265656
75	Fengshunwuhe	China	24445558162255232315343658664434
76	Putao	China	234A6639151213241215331616224433
77	Baozian	China	25451115561245112355454666144656
78	Cubasankeli	Cuba	25466655112412232444234511443435
79	Cuba No.2	Cuba	36467715152414333414243416264635
80	Tailand No.2	Thailand	23455677151325331355453518113434
81	Siji No. 1	Thailand	44885555564444341311343556166655
82	Cuba No.1	Cuba	24454435353424142315333518273636
83	Guinea No1	Guinea	26467715352424133415244568253413
84	Tailand yesheng	Thailand	334A6639351213242415231616124433
85	Africa daxiangya	Africa	23464435152444442314343368223635
86	Guangxi No.4	China	34464558132445331411122266164635
87	Baleys Marvel	America	26361615564614131311343518254636
88	Manzano	America	35667755133311343444244568243456
89	Guangxi No.8	China	27665655352414331315345513343455
90	Miami late	America	56661115132314133414342411224635
91	Liuxiang	Singapore	24445577361225142315453656153336
92	Tumang	China	45686615153414333415233516163656
93	Cuba No.4	Cuba	22464468132214441345342318253636
94	Okrang	Thailand	22464458562214441355342318253636
95	Xiaoqingpi	Thailand	23455688331325331355353518113434
96	Pairi 905	India	22345625132414112311232467563656
97	Herman	America	26457755563444342344231468566656
98	Dazhou	China	22464488352345242315342658354636
99	Kasturi	Indonesia	22256638232213231345133325441613
100	Chocanon	Thailand	44445525152225332511232313564436
101	Dot	America	35465615353314131314122513125655
102	Myahintha	Myanmar	23351655133414231314231518456656
103	Hongmang No.8	America	26461518361215342314242315664435
104	Zill	America	35361115132424133444242316224655
105	Guirewuming	China	45464535132315332315232515464635

106	Pills bury	America	26561645242544131311344515245656
107	13-1	Israel	25465617152311142311345516263336
108	Carrie	America	25341155353312123414231215473656
109	Lengjingdamang	China	25685615353444113414232213225635
110	1515	China	35331515352425133444232416266635
111	Lilley	America	25561111132312141344343544244656
112	Hongmang No.10	America	24464555131225231315342615163445
113	Macheso	Myanmar	276A5555112344131314345513344636
114	Haden	America	36461115132414333424243416264635
115	Rumang	China	36461115112414133414243416264635
116	Guire No.82	China	45464555462315332333232558564645
117	Lvsong	Thailand	24451458561325132345455656113436
118	Hongmang No.11	America	45461655132311133415342368265634
119	Turpentine	America	24465558351215141315235516163445
120	Jinsui	China	25464557132315341335245618244646
121	Tailand hong	Thailand	24441677113414132315235635134636
122	Renong No.2	China	25661155132311331314124511265655
123	Neelum	India	55664415152311333423255518466656
124	20003	China	22455557152315243515235656233433
125	Lvpimang	China	25664435352314343415243568263635
127	20004	China	22464625132344143515343418123413
128	20001	China	24464628162444443315232468153413
129	Hutou	China	22464468152214443355342318253636
130	Mallika	India	25664415333314343315233516463635
131	Edward	India	23461555162411232411444515222456
132	Chuanmang	China	25461158132315343435243588466646
133	Haibao	China	24451525151312121511355658144636
134	Yingzui	Indonesia	22464638132412443515343388253636
135	504	Thailand	24451558351325112355453656163435
136	Guire No.10	China	22464458152214443355342318253636
137	Guixiang	China	25664415112314343435243568265635
138	Thailand No.506	Thailand	25464515132315332415235615664646
139	Yuexi No.1	China	25562218133312143355235666144656
140	Xiaofei	Philippines	27562217552315133314233568153656
141	Langra	India	24485655373414141411234568156656

142	20006	China	22465658662345443555232558224636
143	Huangxiangya	China	25561118333312143355235666144656
144	Nam Klang Wan	Thailand	24445558112255232315343658662434
145	Yiwenda	ChinaTaiwan	25561128233312143345235616144656
146	Yingwu	China	22445677351215233315343615164444
147	Fu No.1	China	55686655333414134413232511246655
148	Yiwen	ChinaTaiwan	45561118133314233355235666124556
149	Hongjingfeng	ChinaTaiwan	25461558562312143314233516144635
150	115	Philippines	22471558352225132311343656563445
151	Chowsa	India	22445577112245343315443618264646
152	Xianggang Hongyu	China	25561118113312143355235644144656
153	1601	China	22464458152214443355342318253636
154	Wenchanghongyu	China	25451557111312143315235516144635
155	Longjing	China	25561117353312142344235616144656
156	Hongjinlong	ChinaTaiwan	45561558362315132314343516244535
157	1317	America	25566615363414343434231518566666
158	Yuwenqingpi	ChinaTaiwan	25561515662314143344342516124656
159	Fu No.3	China	25661825113314133315245568144636
160	Hongxiangya R	China	22486618152345343344242611565645
161	Xingre No.2	China	35361115362424133444352316224655
162	315	China	25561118153312143355235666144656
163	702	Singapore	22684455332444443355233516563635
164	Bambaroo	Australia	46366635552614133415332515254656
165	Ono	America	35561128133414133444232516263656
166	120	Philippines	22444468112345443315342568224636
167	113	Thailand	24455558131325112355255656113436
169	Banganpali	India	27564411353312131314232216443655
170	KRS	Australia	46366635562614133415232515254656
171	R2E2	Australia	56341615362424132315234511255656
172	305	Thailand	24461555162215132315232656164634
173	Alphonso	India	25561128673312141355235666144656
174	Ganhong	ChinaTaiwan	34361155352214132344232411264655
175	Sannian	China	24464555151225132515122336664634
176	Guifei	ChinaTaiwan	34461558382215122344343418663435
177	Fu No.2	China	25561117553312143355234516144656

178	Arumanis	Indonesia	2536461535331134131323555856466 6
179	Dasannian	China	25461158132315331315245618264656
180	Huangyu	Thailand	25451628153412142315235635144636
181	Baodaohuang	Cambodia	25561558362312142315235666144656
182	101	Philippines	22464458332214441355342318253636
183	521	Thailand	22464458112214441355342318253636
184	Thailand Xiaoqingpi	Thailand	22464458352214441355342318253636
185	Bamang	Brazil	225A445838331444135534231826363 6
186	magovar	India	2548151533231111131125555826365 6
187	Panxihong	China	22445658562214142355343518163656
188	Saigon	Vietnam	22685658132415242311342556123435
190	valencia pride	America	25361655132624133414242511564635
191	Spooner	Australia	46446635132414133415232515254656
192	Glenn	America	23681617134414133411344516264656
193	VanDyke	America	2368111156241433234412341126563 5
194	Panyu No.4	China	23341628351344143444342311256645
195	Lvsong 20	Thailand	24341868352325112355455656113436
196	Panyu No.5	China	35581155153444332444342516254635
197	Rosa	Brazil	2536111516141414131134441622363 5
198	Tailand mang	Thailand	24351415132222133515243568266634
199	2203	China	22575557352314341344344618264636
200	Linsheng	China	22475558353414123413242437465535
201	Qiuhong	China	22455515562414123423242268465535
202	2007 Rumang	China	24465655152345343544342466164636
204	Repin No.16	China	23561617133314133411344516264656
205	Zihua	China	25451128562312141355235666144656
206	2001	China	22445558552315242315235656233433
207	2002	China	22485558152412341355342588123346
208	Panyu No.3	China	23455515111425333415342611255645
209	Yuanjiangwanmang	China	34661155162324133444254558124656
210	060620	China	25340055132414133414123411243455
211	063227	China	33451115162322133444252568224655
212	Hubaoya	China	22465558553415243515135656233433
213	060503	China	26464615131212343315235511563656
214	202	China	24464555162425132515122336664634

215	Dongzhenhong	China	45561558351215123344233415664535
216	1506	China	25561118563312143355235666144656
217	Shisheng 1	China	25461118562212141355235666144656
218	Yuemang	Vietnam	22464458352214441355342318253636
219	407	China	22444458352214441355342318253636
220	Duohua	Thailand	44465525562225332511232313564436
221	060123	China	36561155113344133444242216224456
222	Hongwacheng	China	25361428562412141355235666144656
223	Huangxing	China	22455655151345333515234656564645
224	Tailand qing	Thailand	23465688152325331355353518113434
225	20002	China	25564515563415332415235615664646
226	063915	China	23481118562425331355245616226656
227	Tainong No.2	ChinaTaiwan	23445615162424133414242416224535
228	503	Thailand	23561618362414133411144516264656
229	Shisheng 2	China	25661118562612141355235666144656
230	063626	China	24466655112411131344232411565656
231	Xiaohongjinghuang	ChinaTaiwan	45561558353315121344233415664535
233	Mian 8	Myanmar	25465515112244132335233578673656
234	Xingre No.1	China	22341658352414441355342318253636
235	Shisheng	China	25461658132244342344242316264645
236	Xiamao	China	22454458353414441355342318253636
238	301	Thailand	25461117564612143355235666274656
239	060429	China	2346161535224433313244518243636
