# Supplementary material for: Genetic Diversity and Fingerprinting of 231 Mango Germplasm Using Genome SSR Markers
Source: Int J Mol Sci. 2024 Dec 19;25(24):13625. doi: 10.3390/ijms252413625 (PMC11728225; doi:10.3390/ijms252413625)
Supplement: Supplementary file 1 [file ijms-25-13625-s001.zip › Tables S1-S4.pdf]

Table S1. Mango accessions used in the study

Number	Accession	Origin/Source	Number	Accession	Origin/Source
1	Irwin	America	119	Turpentine	America
2	Apple	Malaysia	120	Jinsui	China
3	NamDocMai	Thailand	121	Tailand hong	Thailand
4	Chishu	China	122	Renong No.2	China
5	Arumanis B	Indonesia	123	Neelum	India
6	JinHuang	ChinaTaiwan	124	20003	China
7	Tainong No.1	ChinaTaiwan	125	Lvpimang	China
8	kyo savoy	Thailand	127	20004	China
9	Renong No.1	Australia	128	20001	China
10	Kent	America	129	Hutou	China
11	KenSington	Australia	130	Mallika	India
12	Gundoo	Indonesia	131	Edward	India
13	Graham	America	132	Chuanmang	China
14	Wuyichang	China	133	Haibao	China
15	GeDong R	Indonesia	134	Yingzui	Indonesia
16	Indonesia No.1	Indonesia	135	504	Thailand
17	Sandersha	America	136	Guire No.10	China
18	Indonesia No.2	Indonesia	137	Guixiang	China
19	Baiyu	China	138	Thailand No.506	Thailand
20	Keitt	America	139	Yuexi No.1	China
21	Peach	America	140	Xiaofei	Philippines
22	811	Sri Lanka	141	Langra	India
23	NamDocMai4	Thailand	142	20006	China
24	Zillate	America	143	Huangxiangya	China
25	Mylepania	Australia	144	Nam Klang Wan	Thailand
26	Webber	America	145	Yiwenda	ChinaTaiwan
27	Siji	Thailand	146	Yingwu	China
28	Ruby	America	147	Fu No.1	China
29	Smith	America	148	Yiwen	ChinaTaiwan
30	Julie	the West Indies	149	Hongjingfeng	ChinaTaiwan
31	Tom EJ/C	Israel	150	115	Philippines
32	Lippens	America	151	Chowsa	India
33	Springfels	America	152	Xianggang Hongyu	China

34	Indonesia No.3	Indonesia	153	1601	China
35	Gold Lippens	America	154	Wenchanghongyu	China
36	Beverly	America	155	Longjing	China
37	Sensation	America	156	Hongjinlong	ChinaTaiwan
38	Simmond	America	157	1317	America
39	201003	Thailand	158	Yuwenqingpi	ChinaTaiwan
40	201001	Thailand	159	Fu No.3	China
41	Duncan	America	160	Hongxiangya R	China
42	Florigon	America	161	Xingre No.2	China
43	2020w	China	162	315	China
45	Gangguoshishengzhong	Congo	163	702	Singapore
46	Palmer	America	164	Bambaroo	Australia
47	Eldon	America	165	Ono	America
48	Nam Dok Mai Sitong	Thailand	166	120	Philippines
49	Chenpi	China	167	113	Thailand
50	Anderson	America	169	Banganpali	India
51	Bombay green	India	170	KRS	Australia
52	Ford	America	171	R2E2	Australia
54	1514	China	172	305	Thailand
55	Xiangjiao	Myanmar	173	Alphonso	India
56	Dashehari	India	174	Ganhong	ChinaTaiwan
57	1513	China	175	Sannian	China
58	Panyu No.2	China	176	Guifei	ChinaTaiwan
59	Ai mang	China	177	Fu No.2	China
60	Shengdelong	Myanmar	178	Arumanis	Indonesia
61	Jianmang	Cambodia	179	Dasannian	China
62	Shanlin	ChinaTaiwan	180	Huangyu	Thailand
63	Cuba No.3	Cuba	181	Baodaohuang	Indonesia
64	Xianluo	China	182	101	Philippines
65	Saber	America	183	521	Thailand
66	kwini	Indonesia	184	Thailand Xiaoqingpi	Thailand
67	Hongmangguo	America	185	Bamang	Brazil
68	Dabaiyu	China	186	magovar	India
69	Rjs000l	China	187	Panxihong	China
70	Thailand Carabo	Thailand	188	Saigon	Vietnam

71	Thailand Shengshi	Thailand	190	valencia pride	America
72	Guinea No.2	Guinea	191	Spooner	Australia
73	1030	Indonesia	192	Glenn	America
74	814	Sri Lanka	193	VanDyke	America
75	Fengshunwuhe	China	194	Panyu No.4	China
76	Putao	China	195	Lvsong 20	Thailand
77	Baozian	China	196	Panyu No.5	China
78	Cubasankeli	Cuba	197	Rosa	Brazil
79	Cuba No.2	Cuba	198	Tailand mang	Thailand
80	Tailand No.2	Thailand	199	2203	China
81	Siji No. 1	Thailand	200	Linsheng	China
82	Cuba No.1	Cuba	201	Qiuhong	China
83	Guinea No1	Guinea	202	2007 Rumang	China
84	Tailand yesheng	Thailand	204	Repin No.16	China
85	Africa daxiangya	Africa	205	Zihua	China
86	Guangxi No.4	China	206	2001	China
87	Baleys Marvel	America	207	2002	China
88	Manzano	America	208	Panyu No.3	China
89	Guangxi No.8	China	209	Yuanjiangwanmang	China
90	Miami late	America	210	060620	China
91	Liuxiang	Singapore	211	063227	China
92	Tumang	China	212	Hubaoya	China
93	Cuba No.4	Cuba	213	060503	China
94	Okrang	Thailand	214	202	China
95	Xiaoqingpi	Thailand	215	Dongzhenhong	China
96	Pairi 905	India	216	1506	China
97	Herman	America	217	Shisheng 1	China
98	Dazhou	China	218	Yuemang	Vietnam
99	Kasturi	Indonesia	219	407	China
100	Chocanon	Thailand	220	Duohua	Thailand
101	Dot	America	221	060123	China
102	Myahintha	Myanmar	222	Hongwacheng	China
103	Hongmang No.8	America	223	Huangxing	China
104	Zill	America	224	Tailand qing	Thailand
105	Guirewuming	China	225	20002	China

106	Pills bury	America	226	063915	China
107	13-1	Israel	227	Tainong No.2	ChinaTaiwan
108	Carrie	America	228	503	Thailand
109	Lengjingdamang	China	229	Shisheng 2	China
110	1515	China	230	063626	China
111	Lilley	America	231	Xiaohongjinghuang	ChinaTaiwan
112	Hongmang No.10	America	233	Mian 8	Myanmar
113	Macheso	Myanmar	234	Xingre No.1	China
114	Haden	America	235	Shisheng	China
115	Rumang	China	236	Xiamao	China
116	Guire No.82	China	238	301	Thailand
117	Lvsong	Thailand	239	060429	China
118	Hongmang No.11	America			

Table S2. Primer sequences used in this study

Primer code	Repeat motif	Primer sequence(5'-3')	5'Fluorescent Labeling	Expected product (bp)
G33	CCA(8)	F: GCCATAAGTATTGCGAGCGC	HEX	187
		R: ACGCCTAGTGGTGAAGAAGC		
G111	ACC(15)	F: TGGCGGGAGATTCCAAATCG	FAM	122
		R: GTTGAGCTTGCACATGGTGG		
G216	TTA(7)	F: AGATCCCTGCCAAAAGCTCC	FAM	133
		R: CAGCATGGAGGAGAATGTTAACG		
G304	AAC(7)	F: TCTTCCAACTCCTCAACTGAGC	FAM	120
		R: AGAGTGAGAACCTGGCAAGC		
G386	GGAACA(5)	F: CGGCGAGTTGAGAAACAAAGG	HEX	157
		R: TCCACAGAACACCAAACTCCC		
G440	TTG(10)	F: AACGAAAACCAGGCATCACC	HEX	131
		R: GTTTATGGCAATTGCAGCAGC		
G573	TGACTG(4)	F: GACCCACAGTGATTGTCTTTCC	HEX	137
		R: CATGGTTTTCGTGTTGGGCC		
G584	CCT(8)	F: AGACACTCCACAAGCTGTGC	FAM	114
		R: TTGGAGTGTTGGAGTTGCCC		
G589	TGCTGG(4)	F: TTGATCCTTGAGCCTGAGGG	ROX	173
		R: AACTCAAGCACCAGGACTGC		
G676	TTCTAG(4)	F: CCCAGTTGAAAATATGGGTTCCC	ROX	194
		R: TCCACCGCTGAAACAACTCG		
G744	ACA(7)	F: AGATGGACGGTTGAGGTTGC	HEX	166
		R: TTCCCCCTTAAATGGCCACC		
G824	AAT(7)	F: GCAAGCGCATGAAGTATGACC	FAM	110
		R: TGCTCCCCAATTTCCATTTTGC		
G833	CCA(8)	F: ACCATCATCACCATTCCCCC	HEX	147
		R: ACTGCCACTCTGTTCCTTCG		
G886	GCCATC(4)	F: GCCTGTGCATTCATCAACCG	ROX	148
		R: ACCCATTGGAGATGGTCTGC		
G1028	AAG(10)	F: ATCCGGTGCTGATCATCAGG	HEX	122
		R: TCCTGGATGAAACCTTGGAGC		
G1077	GAC(7)	F: AGTTTGGCACCTCTTGGAGC	HEX	134
		R: TTCCTCTTCACCCGAACTCG		
G1097	TGGTGC(4)	F: TGGAAAGCCTTTGCAATCCC	HEX	151
		R: TCAATCTTGGGACCAGCACC		
G1134	TGCTTC(4)	F: CACATTGTCCCAACATGCCC	FAM	127
		R: GAGCAGCACACAACTTGAGG		
G1151	CAA(7)	F: GTCAGATGTTGTCCGCATGC	ROX	204
		R: TCATCGTCCATTCCTGTGCC		
G1212	GTC(7)	F: TGTCAATCAGGTCTCGTCGG	HEX	148
		R: TCGATGAGTCAATCCACCGC		
G1234	CTCTCC(5)	F: TCGTTTTTCCCTTCCTTCCCC	FAM	103
		R: AGGGGAAGCACAGAGAATCG		
G1268	GAGCAT(4)	F: ACCTCATGAGTGCCGTAACG	ROX	167
		R: TAAAGCCACAACGACATCGC		
G1342	GAGGTT(4)	F: TGGACTCGACATCCTGAAGC	ROX	170

		R: CTTCACCGCTTTGTCAAGGG		
G1352	TCA(10)	F: GCTTGCAGCCATGAAGAACC	HEX	147
		R: AGCTGATAATCTGATGGAGCTGG		
G1360	CTT(7)	F: GCAACTCAACTGGTTCAGTGC	HEX	136
		R: AATGGAGGCTTCTGGTACGC		
G1382	TGC(7)	F: TCCACGGTGGATGATGTAGC	FAM	118
		R: GAAGATTCAGCAAGCCAGACG		
G1424	TCA(7)	F: AGGATTCCATCACCGAAGCC	FAM	116
		R: CCTTTTGTTGCAGGGTTGGG		
G1450	TTCG(6)	F: ATGCTTCACCAAAACGACGC	FAM	114
		R: CAAACAGAGCAACAGAACGGG		
G1454	ATA(10)	F: AAAAGGAGGACGGTTTGGGG	FAM	133
		R: GTACCCACCAAATTAGGCGC		
G1527	CTG(7)	F: CACCCGTCTTGAACCTCTCC	ROX	192
		R: TCAGGCAAATGAGTGCTGGG		
G1617	ACA(8)	F: CAAACACGAAGACGACGACG	FAM	126
		R: TCGCCGTTGTCCATATTCCC		
G1666	TGA(9)	F: TATAACGCTGGACACGACGC	HEX	128
		R: GCCAAACCTTGACTTCATTACAGG		
G1745	GAA(10)	F: TGAGTGTGCCCAATGTTTGC	HEX	135
		R: CAAGCCCAAACCCAATCACC		
G1783	GTG(7)	F: GGAAGTTGCAGCAATAAGGGG	HEX	133
		R: ACCACACATCCTGAGCAACC		

Table S3. SSR counts distribution for each type

Type	Counts	Percent (%)
Mono	54520	49.94
Di	29830	27.32
Tri	11327	10.37
Tetra	9016	8.26
Penta	3347	3.07
Hexa	1137	1.04

Table S4. Distribution of maximum Q-values for GroupIand GroupII

population	Number of each group	Number(%)	
		Q>=0.6	Q>=0.9
GroupI	104	84(80.77%)	42(40.38)
GroupII	127	112(88.19)	66(51.97%)
Total	231	196(84.85%)	108(46.75%)
